# IL-21-dependent Ly6C^+^Ly6G^+^CD4^+^ T cells found in lung enhance macrophages function against *Actinobacillus pleuropneumoniae* infection in mice

**DOI:** 10.1038/s41420-025-02742-z

**Published:** 2025-10-06

**Authors:** Chuntong Bao, Xuan Jiang, Yanyan Tian, Wenjing Wang, Jiameng Xiao, Baijun Liu, Peiru Chen, Ziheng Li, Jiuyan Li, Junhui Zhu, Tamim Abdelaal, Dexi Chen, Na Li, Liancheng Lei

**Affiliations:** 1 State Key Laboratory for Diagnosis and Treatment of Severe Zoonotic Infectious Diseases, Key Laboratory for Zoonosis Research of the Ministry of Education, Institute of Zoonosis, and College of Veterinary Medicine, Jilin University, Changchun, 130062 China; 2 Beijing Institute of Hepatology, Beijing Youan Hospital, Capital Medical University, 100069 Beijing, China; 3 The Laboratory Department of First Hospital, Jilin University, Changchun, China; 4 Leiden Computational Biology Center, Leiden University Medical Center, Leiden, The Netherlands; 5 Department of Pattern Recognition and Bioinformatics Group, Delft University of Technology, Delft, the Netherlands

**Keywords:** Acute inflammation, Cell death

## Abstract

IL-21/IL-21R signaling is crucial in various immune diseases and cellular development, however, its role in bacterial pneumonia remains unclear. Here, IL-21R knockout (IL-21R^−^^/−^) mice were more susceptible to *Actinobacillus pleuropneumoniae* (APP) than wild-type (WT) mice. High-dimensional mass cytometry analysis revealed that IL-21R deficiency inhibited neutrophil activation, decreased the numbers of monocytes and proinflammatory macrophages, and augmented the defective CD3^low^ T cells in the lungs. Intracellular cytokine staining showed decreased IFN-γ/TNF-α/IL-6 production in IL-21R^−^^/−^ mice, particularly in CD8⁺ T cells. Furthermore, a previously unrecognized Ly6C^+^Ly6G^+^CD4^+^ T cell subset emerged only in the lungs of WT mice post-APP infection, which was in an activated status with stronger secretion capacities of IL-10, IL-21, granzyme B, and perforin by flow cytometry. These cells polarized macrophages into M2- or M1- phenotype without/with infection, respectively, and enhanced proliferation, phagocytosis, and macrophage extracellular traps/ROS-mediated bactericidal activity of macrophages against-APP, *Klebsiella pneumoniae*, or *Escherichia coli* infection. Thus, our study demonstrated that IL-21 drives the differentiation of neutrophils, monocytes, and macrophages into pro-inflammatory subsets. IL-21-induced Ly6C^+^Ly6G^+^CD4^+^ T cells cooperate with macrophages to enhance bacterial clearance, providing a promising target for preventing bacterial pneumonia.

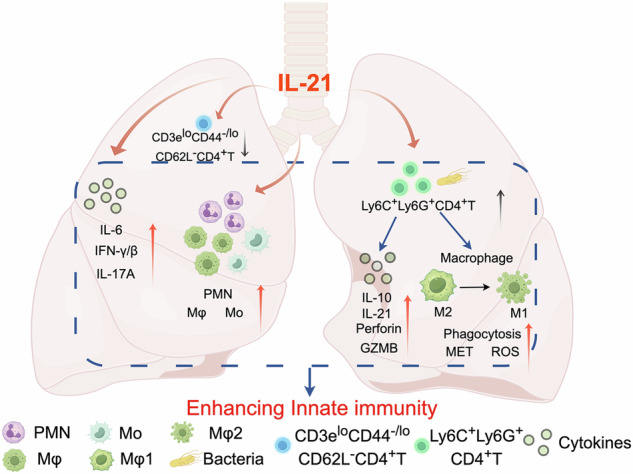

## Introduction

Pneumonia is a severe lung infection caused by pathogens, posing a significant threat to human and animal health. Before the COVID-19 pandemic, pneumonia caused up to 50,000 deaths annually in the U.S. [1–5]. While vaccines and antibiotics remain primary preventive and therapeutic measures, antimicrobial resistance and the lack of broad-spectrum vaccines highlight the need for alternative treatments. A better understanding of the lung immune system may lead to novel therapeutic strategies. In recent years, with the advent of single-cell technologies such as high-throughput mass cytometry, several immune cell subsets in lungs with important immune regulatory functions have been discovered and even entered clinical applications. For example, sB-1a B cells, which secrete high-affinity IgM, provide protection against pneumococcal pneumonia [6–8] and mitigate lung inflammation by producing IL-10, IL-35, and other anti-inflammatory factors [9–11]. These cells are now considered targets for bacterial and viral pneumonia treatment, including COVID-19 [12].

*Actinobacillus pleuropneumoniae* (APP) causes porcine pleuropneumonia, a highly contagious and lethal lung disease of pigs, and is an important pathogen threatening the pig breeding industry [13–15]. APP has a strong tropism for the lungs, and it is often used to make an experimental pneumonia model. APP-infected mice exhibit lung pathology similar to pigs, making them a valuable tool for pneumonia research and drug evaluation [16–19].

IL-21, primarily produced by CD4^+^ T and NKT cells, exerts broad immunomodulatory effects via IL-21R, which is widely expressed on lymphocytes, myeloid, and epithelial cells [20, 21]. Our previous research found that IL-21 significantly increased at 24 h post-infection in the bronchoalveolar lavage fluid of APP-infected piglets, but not in sera [22], suggesting its localized role in lung immunity. Previous work reveals that IL-21 modulates immune responses by inhibiting cytokine production in macrophages [23] and dendritic cells [24], supporting iNKT and ILC3 maintenance in fungal infections [25], and cooperating with type I interferons to eliminate Staphylococcus aureus [26]. However, it can also suppress Th1 and Th17 responses, exacerbating *Chlamydia muridarum* lung infections [27]. However, the immunological effects of IL-21 on the lung immune response are still poorly understood during acute bacterial pneumonia.

To study the roles of IL-21/IL-21R signaling in bacterial pneumonia, we used APP as a model bacterium to establish an acute lung infection model in wild-type (WT) and IL-21R knockout (IL-21R^−^^/−^) mice, and applied mass cytometry and flow cytometry to explore the roles of IL-21/IL-21R in APP infection. With the help of high-throughput mass cytometry, the influence of IL-21/IL21R signaling on the composition and function of lung immune cells was analyzed to determine the mechanisms and roles of key immune subsets in APP-induced pneumonia. We found that IL-21/IL-21R signaling enhances lung defense against APP by promoting early inflammatory responses. Furthermore, a previously unknown CD4^+^ T cell subset expressing Ly6C and Ly6G on the cell surface, which had a strong ability to secrete IL-10, IL-21, perforin, and granzyme B, was dependent on IL-21 and found only in WT mice lungs infected with APP, *Klebsiella pneumoniae* or *Escherichia coli*. Moreover, these cells promoted the proliferation of macrophages, polarized macrophages from the M2- to M1- phenotype post-infection and increased the phagocytic and bactericidal function (macrophage extracellular traps (METs) or reactive oxygen species (ROS) of macrophages, contributing to the host defense against bacterial infection. Thus, our data lay the foundation for the establishment of novel pneumonia prevention and treatment methods based on the host immune response.

## Results

### IL-21/IL-21R is critical for the defense to APP infection in mice

**Fig. 1:**
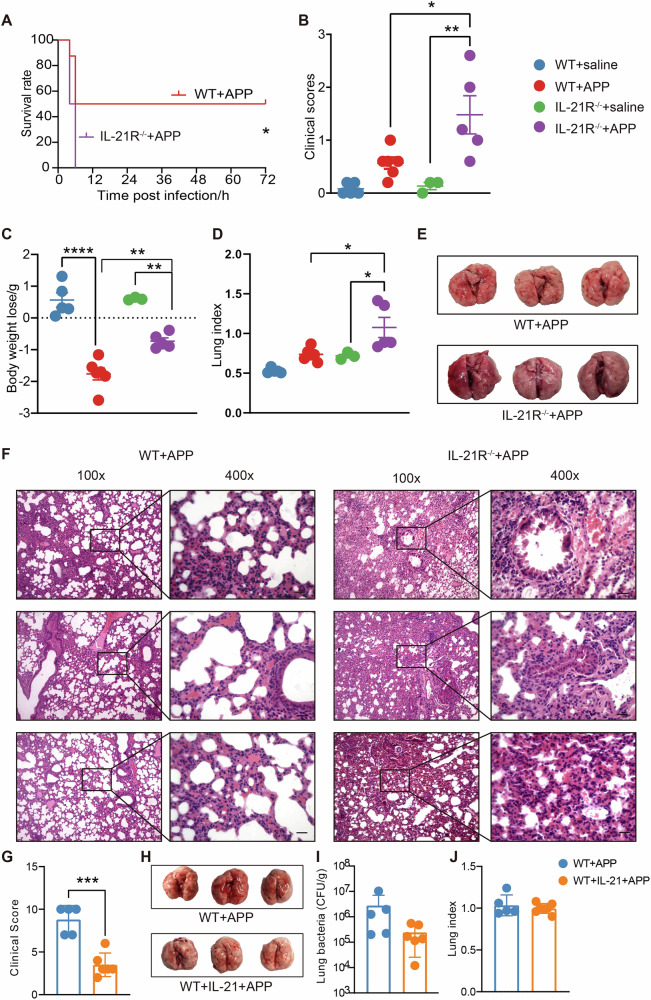
Assessment of the effect of the IL-21/IL-21R signaling pathway on mice infected with APP. **A** The survival rate of WT and IL-21R^−^^/−^ mice were observed within 72 h post APP (3.2 ×106 CFU/mouse) infection. **B**–**F** WT and IL-21R^−^^/−^ mice were infected with APP intranasally (1 ×106 CFU/mouse), the (**B**) clinical scores, **C** weight loss, **D** lung index, **E** lung macroscopic pathological changes, and **F** HE staining results of pathological sections of lungs were recorded 12 h after infection. **G**–**J** After IL-21 supplement, the (**G**) clinical score, **H** lung macroscopic pathological changes, **I** lung bacterial burden, and **J** lung index were recorded 12 h after APP infection. Scale bar = 100 μm *p < 0.05; **p < 0.01; ***p < 0.001; ****p < 0.0001, using Mann–Whitney U test. Error bars show means ± SEM.

To explore the role of IL-21 in bacterial pneumonia, APP was used as a model bacterium to establish infection in both wild-type (WT) and IL-21R knockout (IL-21R^−^^/−^) mice. Compared with WT mice, IL-21R^−^^/−^ mice were extremely susceptible to APP infection (3.2 ×106 CFU/mouse), all mice dying within 6 h after infection (Fig. 1A). Due to the high susceptibility of IL-21R^−^^/−^ mice to APP, a lower dose of APP (1 ×106 CFU/mouse) was used to evaluate the differences caused by IL-21R between WT and IL-21R^−^^/−^ mice. IL-21R^−^^/−^ mice displayed more severe clinical manifestations such as malaise, loss of appetite, coarse back hair, and abdominal breathing (Fig. 1B). Compared to WT mice, the body weight of IL-21R^−^^/−^ mice was also reduced significantly post-infection (Fig. 1C), while the lung index only increased significantly in IL-21R^−^^/−^ mice (Fig. 1D). Moreover, IL-21R^−^^/−^ mice had worse lung injury with swelling, hemorrhage (Fig. 1E), severe alveolar wall thickening, and alveolar expansion with cell debris and red blood cells, indicating more severe lung pathological damage (Fig. 1F). To further explore the IL-21-drived defense immune response to APP infection, IL-21 was pre-administered to examine its function in the mice model of APP-induced pneumonia. The addition of IL-21 considerably reduced clinical symptoms (Fig. 1G), lessened lung injury (Fig. 1H), and decreased lung bacterial load (Fig. 1I), but there was no discernible difference in lung index (Fig. 1J). Thus, our data indicate that IL-21/IL-21R signaling was critical for the defense against APP infection in mice.

### Mass cytometric analysis reveals IL-21-induced distinct myeloid cell spectrum in mouse lung post-APP infection

**Fig. 2:**
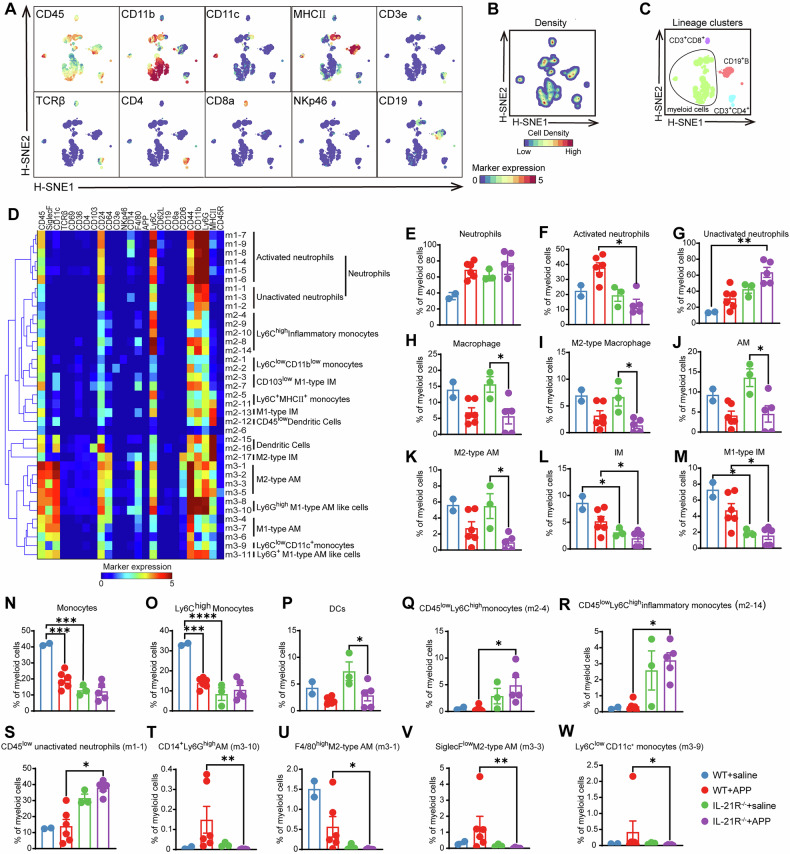
Identification of phenotypically distinct clusters in myeloid cell compartment in the mouse lungs. **A**–**C** Analysis of the whole immune system across all the mouse lung tissues using mass cytometry (*N* = 17 samples, including 1,096,882 cells). **A** H-SNE represents the overall composition of the lung immune system. Expression profile of the indicated markers. **B** Density characteristics of the embedded cells. **C** A H-SNE plot shows the cluster partitions of major immune cell lineages in the lung. **D** A phenotypic heatmap (scale from blue to red) shows the median marker expression values and hierarchical clustering of each cell cluster in Fig. S2. **E**–**W** The bar plots show the proportion of the indicated cell clusters identified in panel D in each group. AM alveolar macrophages. *p < 0.05; **p < 0.01, using Mann-Whitney U test. Error bars show means ± SEM.

To explore the differences in the lung immune cell composition between WT and IL-21R^−^^/−^ mice after APP infection, high-dimensional mass cytometric analysis was applied to identify the major immune cell lineages in mouse lungs (Fig. 2A, B), where four major immune lineages were identified with distinct phenotypes at the overall level, namely CD19^+^ B cells (CD3^-^CD19^+^), CD3^+^CD4^+^ T cells (CD19^-^CD3^+^TCRβ^+^CD4^+^), CD3^+^CD8^+^ T cells (CD19^-^CD3^+^TCRβ^+^CD8^+^), and myeloid cells (CD3^-^TCRβ^-^CD19^-^NKp46^-^CD11b^+^/CD11c^+^) (Fig. 2C). However, there were no significant changes in the proportion of the above four major immune cell lineages between IL-21R^−^^/−^ and WT mice (Supplementary Fig. 1A–D).

Further, the reason why IL-21R^−^^/−^ mice were more susceptible to APP infection was dissected from the perspective of innate immune cells. Myeloid cells (789,441 cells) were first selected at the overall level and embedded into the second level of H-SNE analysis. According to the density characteristics of the embedded cells, three groups of myeloid cells were identified and a t-SNE algorithm was computed to further exploit the heterogeneity in these three meta-clusters, separately (Supplementary Fig. 2A–I). Based on the marker expression profiles and cell density, 37 phenotypically distinct myeloid cell clusters in total were identified using Gaussian Mean-Shift clustering, reflecting the unprecedented heterogeneity of myeloid cell compartment (Supplementary Fig. 2). After hierarchically clustering, these clusters were further grouped into 14 main cell populations (Fig. 2D), namely, neutrophils, including both activated neutrophils highly expressing CD11b, Ly6G, CD24, and CD44, and unactivated neutrophils with the CD44^low^Ly6C^low^CD24^low^ phenotype; Ly6C^high^ inflammatory monocytes; Ly6C^low^CD11b^low^ monocytes; CD103^low^ M1-type interstitial macrophages (IM); Ly6C^+^MHCⅡ^+^ monocytes; M1-type IM; CD45^low^ dendritic cells; dendritic cells; M2-type IM; M2-type alveolar macrophages (AM); M1-type AM; Ly6G^high^ M1-type AM-like cells; Ly6C^low^CD11c^+^ monocytes and Ly6G^+^ M1-type AM-like cells.

We next statistically analyzed the proportions of the above 14 types of cell populations in each group and found that there were no significant changes in the total number of neutrophils before and after the IL-21R deletion (Fig. 2E). However, after APP infection, the absence of IL-21R significantly inhibited the activation of lung neutrophils (Fig. 2F) but promoted the significant accumulation of the unactivated neutrophils in the lung (Fig. 2G). After APP infection, macrophages, an essential pulmonary innate immune cell, dramatically decreased in IL-21R defective mice, although the proportions of macrophages and M2-type macrophages in the WT mice did not alter significantly (Fig. 2H, I). The two different types of macrophages present in the lung are AMs and IMs. M2-type AMs were responsible for the decline in IL-21R^−^^/−^ mice, according to further examination of the AM subsets (Fig. 2J, K). Additionally, both at the fundamental level and post-infection, the proportion of total IM and the M1-type IM was significantly reduced in IL-21R^−^^/−^ mice (Fig. 2L, M). In the lungs of WT mice, the APP infection significantly reduced monocytes, particularly Ly6C^high^ inflammatory monocytes. IL-21R^−^^/−^ mice had weaker inflammatory responses in their lungs than WT mice because the absence of IL-21R directly caused a considerable decrease in the number of monocytes and Ly6C^high^ inflammatory monocytes in the lung at the basic level and kept it low following APP infection (Fig. 2N, O). There was no discernible difference in the dendritic cell population between WT and IL-21R^−^^/−^ mice before and after infection. However, there was a significant drop in the dendritic cell population in IL-21R^−^^/−^ mice after APP infection (Fig. 2P).

IL-21R deficiency also resulted in a higher proportion of cell clusters with low expression of CD45 in the lung at basic level and the APP infection markedly increased the proportion of these cells in IL-21R^−^^/−^ mice, including CD45^low^Ly6C^high^ monocytes (m2-4, m2-14), and CD45^low^ unactivated neutrophils (m1-1) (Fig. 2Q, S), further indicating that IL-21 may affect immune cell activation. Additionally, CD14^+^Ly6G^high^AM (m3-10), F4/80^high^ M2-type AM (m3-1), SiglecF^low^ M2-type AM (m3-3), and Ly6C^low^CD11c^+^ monocytes (m3-9) were decreased significantly in IL-21R^−^^/−^ mice compared to WT mice post-APP infection (Fig. 2T, W), indicating IL-21 may affect the differentiation of macrophages.

In conclusion, our data provide evidence for the existence of IL-21-associated immune clusters and indicate that IL-21R deficiency could inhibit the differentiation of neutrophils, monocytes, and macrophages into pro-inflammatory subsets, leading to the increase in susceptibility of IL-21R^−^^/−^ mice to APP infection.

### IL-21R deficiency leads to insufficient proinflammatory cytokine expression in the lung

**Fig. 3:**
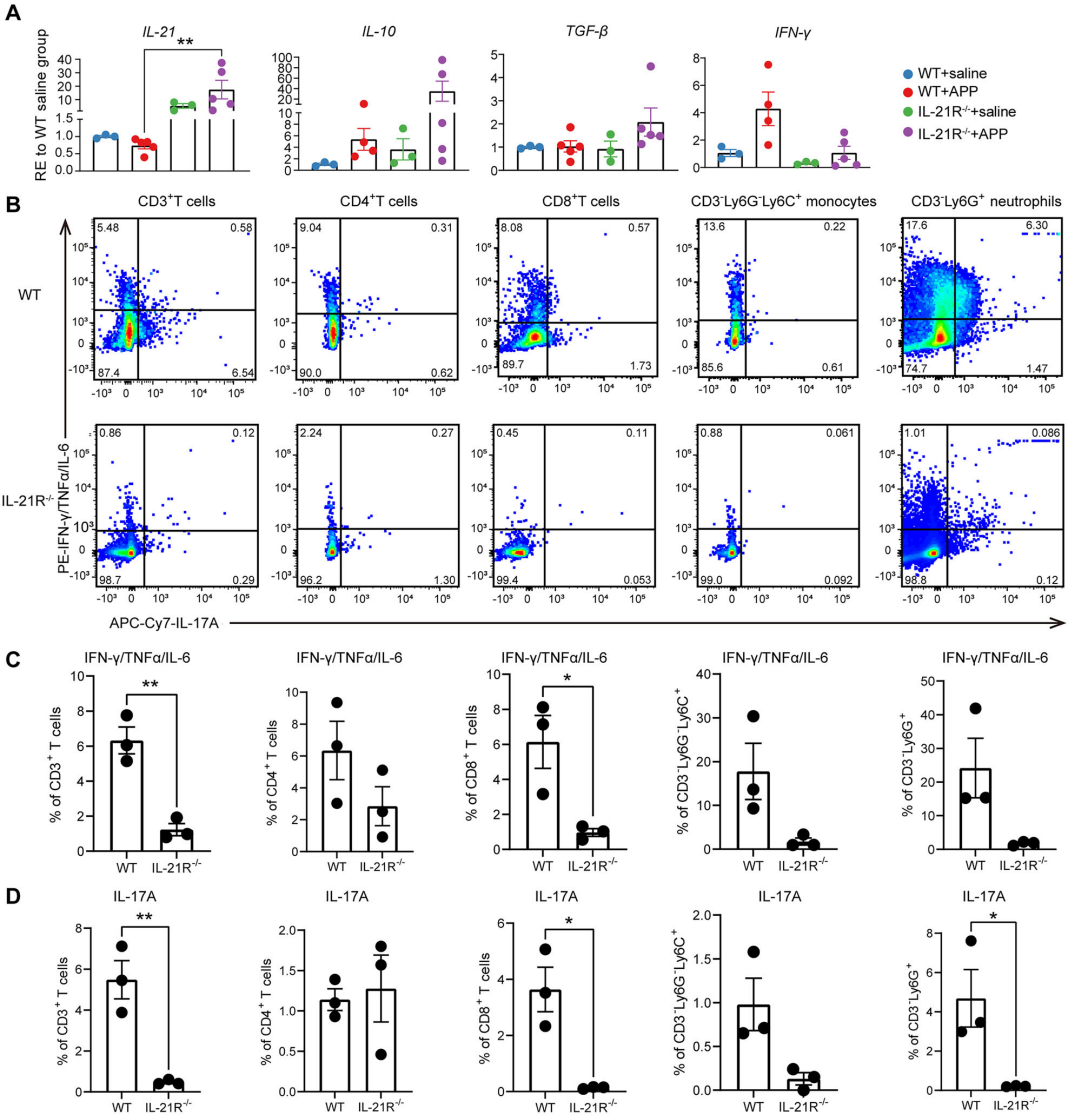
Detection of cytokines in the lung tissues and immune cells of WT and IL-21R^−^^/−^ mice 12 h after APP infection. **A** mRNA expression of *IL-21, IL-10, TGF-β*, and *IFN-γ* in the lung tissues of WT and IL-21R^−^^/−^ mice at 12 h post-APP infection by RT-qPCR. **p < 0.01, using Mann-Whitney U test. Error bars show means ± SEM. **B**–**D** Secretion of pro-inflammatory cytokines IFN-γ/TNF-α/IL-6 and IL-17A of CD3^+^ T cells, CD4^+^ T cells, CD8^+^ T cells, CD3^-^Ly6G^-^Ly6C^+^ monocytes, and CD3^-^Ly6G^+^ neutrophils in the lungs of WT and IL-21R^−^^/−^ mice. **B** The representative gating strategies of lung immune cells in WT and IL-21R^−^^/−^ mice are shown. **C**, **D** The secretion of pro-inflammatory cytokines (**C**) IFN-γ/TNF-α/IL-6 and (**D**) IL-17A in the lung CD3^+^ T cells, CD4^+^ T cells, CD8^+^ T cells, CD3^-^Ly6G^-^Ly6C^+^ monocytes, and CD3^-^Ly6G^+^ neutrophils were statistically analyzed. *p < 0.05; **p < 0.01, using unpaired T test. Error bars show means ± SEM.

To further determine the reasons for IL-21R^−^^/−^ mice being extremely susceptible to APP, RT-qPCR was used to analyze the variations in cytokine expression between WT and IL-21R^−^^/−^ mice in the lung microenvironment at a transcription level. The results showed that *IL-21* was highly expressed and *IL-10 increasing trend* in IL-21R^−^^/−^ mice after APP infection, while *TGF-β* and *IFN-γ* did not respond to APP infection as WT mice did (Fig. 3A). Subsequently, flow cytometry was applied to further detect the extent of secretion of pro-inflammatory cytokines (IFN-γ/TNF-α/IL-6) and IL-17A in CD3^+^ T cells, CD4^+^ T cells, CD8^+^ T cells, CD3^-^Ly6G^-^Ly6C^+^ monocytes, and CD3^-^Ly6G^+^ neutrophils in the lungs of WT and IL-21R^−^^/−^ mice post-APP infection. The production of pro-inflammatory cytokines (IFN-γ/TNF-α/IL-6) in T cells, monocytes, and neutrophils had a downward trend in the lungs of IL-21R^−^^/−^ mice, especially in CD8^+^ T cells (Fig. 3B, C). IL-21R deficiency also reduced the IL-17A production in CD8^+^ T cells, neutrophils, and monocytes, however, it did not affect the production of IL-17A in CD4^+^ T cell populations (Fig. 3B, D). Therefore, the data indicated that the IL-21R deficiency may decrease the ability to resist APP infection through weakening the lung cytokine immune response.

### IL-21/IL-21R signaling shapes the composition of unrecognized Ly6C^+^Ly6G^+^ T cells and reduces defective CD3^low^ T cells in the mouse lung post-APP infection

**Fig. 4:**
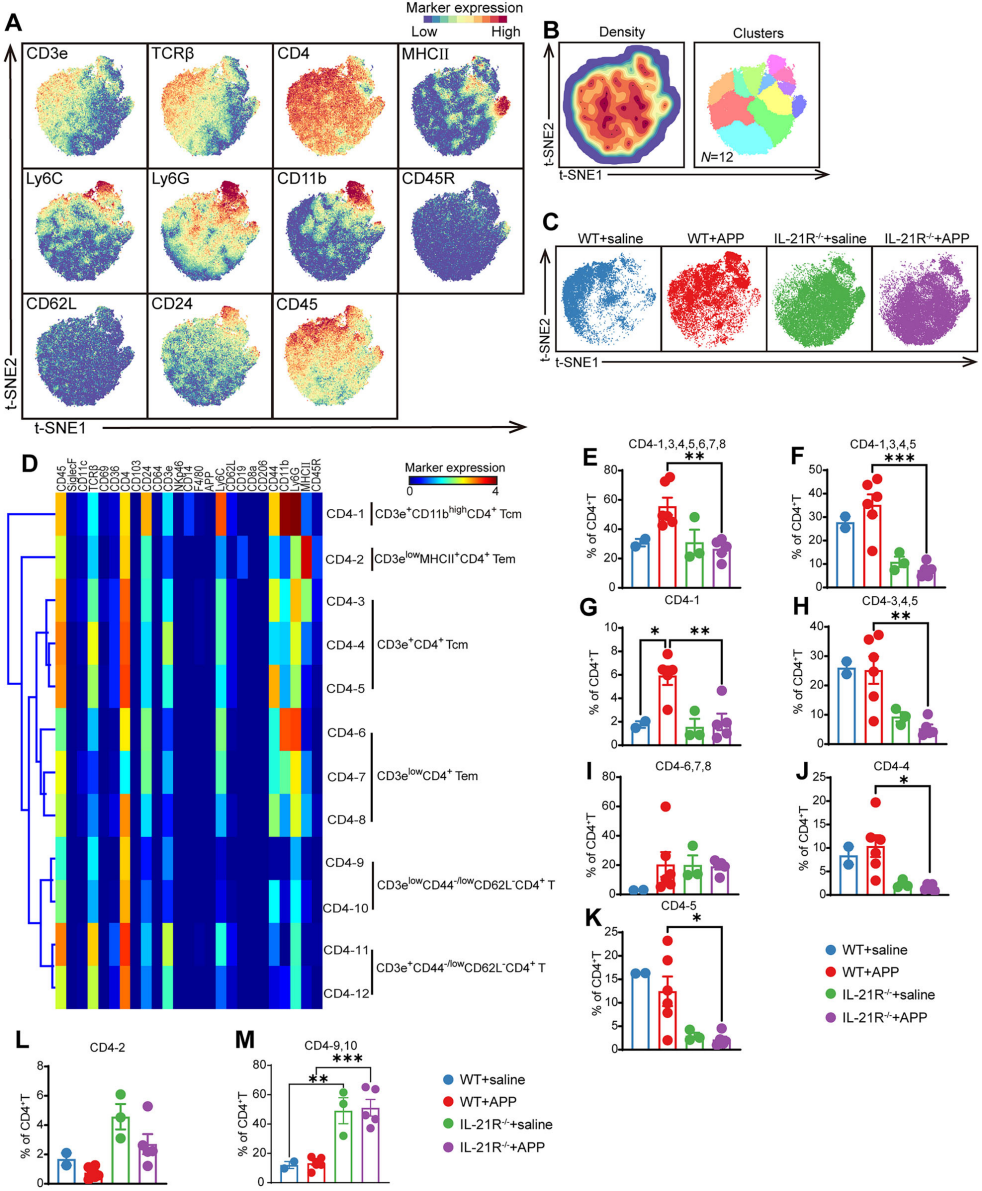
Identification of CD4^+^ T cell clusters in WT and IL-21R^−^^/−^ mice lungs infected with APP. **A** A H-SNE embedding of 91,586 CD4^**+**^ T cells. Colors show the expression of the indicated markers. **B** A density map of CD4^**+**^ T cell compartment (left panel) and cluster partitions in different colors. (Right panel). **C** t-SNE plots showing the cell distribution in each indicated group (**D**) Phenotypic heatmap (scale from blue to red) shows the median marker expression values and hierarchical clustering of each cell cluster in CD4^**+**^ T cells. **E**–**M** Statistical analysis of the proportion of indicated clusters in CD4^**+**^ T cells in (**D**).*p < 0.05, using Mann-Whitney U test. Error bars show means ± SEM.

IL-21 is mainly produced by CD4^+^ T cells, while its receptor IL-21R is widely distributed among CD4^+^ T, CD8^+^ T, and even CD19^+^ B cells. Therefore, we next determined the influence of IL-21R on these lymphoid cells. Taking the CD4^+^ T cell compartment as an example, we explored the clusters that were related to IL-21/IL-21R signaling in APP-infected lungs using a similar pipeline to the myeloid cell population (Fig. 4). First, a t-SNE dimensional reduction analysis was performed on CD4^+^ T cell population (91,586 cells), in which 12 CD4^+^ T cell clusters with distinct phenotypes were identified and clustered into 6 metagroups (Fig. 4A–D). Strikingly, Ly6C and Ly6G expression was present in multiple memory T cell clusters (CD4-1, 3, 4, 5, 6, 7 and 8), and the subset was significantly reduced in IL-21R^−^^/−^ mice compared to the WT mice post-infection (Fig. 4D, E). Furthermore, this reduction was attributable to central memory T cells (Tcm) (CD4-1,3,4,5), specifically CD3e^+^CD11b^high^Ly6G^high^CD4^+^ Tcm (CD4-1) and CD3e^+^CD4^+^ Tcm (CD4-3,4,5), but not to effector memory T cells (Tem), specifically CD3e^low^CD4^+^ Tem (CD4-6,7,8) (Fig. 4F–I). In order to further examine the variations in the percentage of these three CD3e^+^CD4^+^ memory T cell clusters in each group, MHCII^-^ clusters CD4-4 and CD4-5 contributed significantly to WT mice post-APP infection (Fig. 4J, K). It was noteworthy that Ly6G is a hall marker for neutrophils. Its expression on T cells, however, has not yet been documented. Interestingly, T cells with limited number of TCR/CD3 receptor, including CD3e^low^CD4^-^Tem (CD4-6,7,8), CD3e^low^MHCII^+^CD4^+^ T cells (CD4-2), and CD3e^low^CD44^-/low^CD62L^-^CD4^+^T cells (CD4-9, 10) were dominant in the IL-21R^−^^/−^ mice with the latter showing significant changes following statistical analysis (Fig. 4I, L-M), suggesting the defective T cell responses to stimulus after IL-21R deficiency.

A similar analysis was also performed on the CD8^+^ T cell compartment, and three phenotypically distinct clusters were identified, namely CD3e^low^CD8^+^Tem (CD8-1,2,3,4,5), CD3e^+^CD8^+^Tem (CD8-7,8,9,10) and CD3e^+^CD44^-^CD62L^-^CD8^+^ T (CD8-11,12,13,14) (Supplementary Fig. 3A–D). Interestingly, Ly6C and Ly6G coexpression T cell subsets such as CD8-2, CD8-5 and CD8-8 were also identified in the CD8^+^ T cell population (Supplementary Fig. 3D). Moreover, the proportions of these cells in the lungs of IL-21R deficient mice infected with APP were significantly less than those of WT mice (Figure S3E-G), indicating the unrecognized Ly6C^+^Ly6G^+^CD8^+^ T cells were also IL-21-dependent. Moreover, the majority of CD8^+^ T cells in the lung of IL-21R^−^^/−^ mice were TCRβ^low^CD3e^low^ (CD8-6) (up to 46.07% in uninfected mice and 48.69% in APP-infected mice), while the CD8^+^ T cells in the WT mice were TCRβ^+^CD3e^+^ (Supplementary Fig. 3I).

Altogether, these data indicate that IL-21 inhibited the accumulation of CD3e^low^ T cells with defective responses and provide evidence of the existence of unrecognized IL-21/IL-21R signaling dependent Ly6C^+^Ly6G^+^CD4^+^ T and Ly6C^+^Ly6G^+^CD8^+^ T cells in the lungs of WT mice after APP infection.

### Ly6C^+^Ly6G^+^CD4^+^ T cells are in an activated status with a stronger secretion ability of IL-10, IL-21, perforin, and granzyme B

**Fig. 5:**
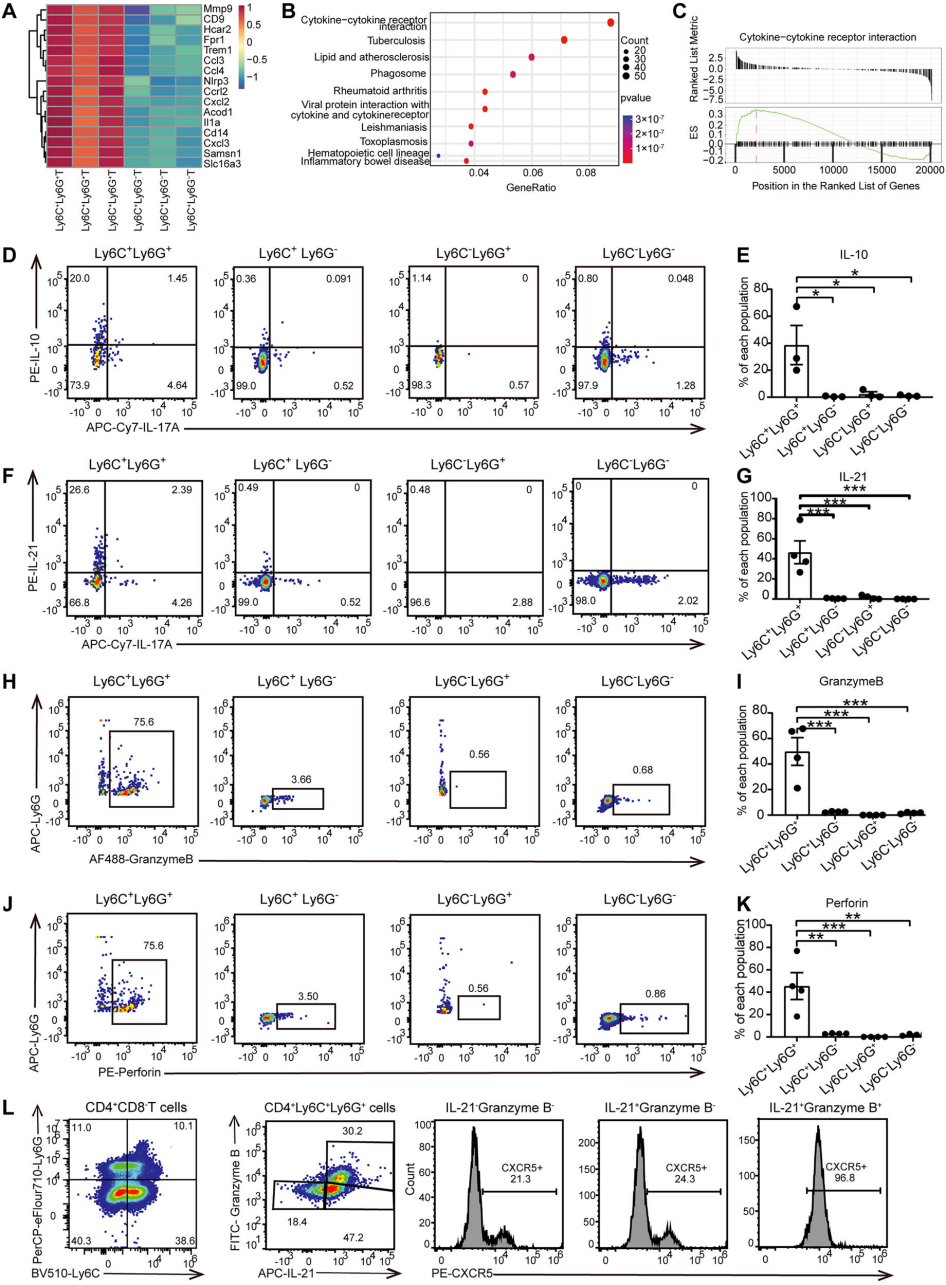
Cell characteristics analysis of Ly6C^+^Ly6G^+^CD4^+^ T cells. **A**–**C** The transcriptome analysis of Ly6C^+^Ly6G^+^CD4^+^T and Ly6C^+^Ly6G^-^CD4^+^T cells. **A** The heatmap showing the expression value of indicated genes, **B** the top 10 KEGG Pathway enrichment in Ly6C^+^Ly6G^+^CD4^+^T cells, **C** GSEA plots of cytokine-cytokine receptor interaction in Ly6C^+^Ly6G^+^CD4^+^T compared to Ly6C^+^Ly6G^-^CD4^+^T cells. **D**–**L** The representative biaxial plots and statistics analysis of (**D**, **E**) IL-10, (**F**, **G**) IL-21, (**H**, **I**) granzyme B, and (**J**, **K**) perforin secretion in Ly6C^+^Ly6G^+^CD4^+^ T cells, Ly6C^+^Ly6G^-^CD4^+^ T cells, Ly6C^-^Ly6G^+^CD4^+^ T cells, and Ly6C^-^Ly6G^-^CD4^+^ T cells, respectively. **L** The expression of CXCR5 in IL-21^-^Granzyme B^-^, IL-21^+^Granzyme B^-^ and IL-21^+^Granzyme B^+^ cells of CD4^+^Ly6C^+^Ly6G^+^ cells. *p < 0.05; **p < 0.01; ***p < 0.001, using Mann-Whitney U test. Error bars show means ± SEM.

To understand the characteristics of Ly6C^**+**^Ly6G^+^ T cells, we sorted Ly6C^**+**^Ly6G^**+**^CD4^**+**^ T cells and Ly6C^**+**^Ly6G^**-**^CD4^**+**^ T cells to perform bulk RNA-seq from APP-infected mouse lungs. Compared to Ly6C^**+**^Ly6G^**-**^CD4^**+**^ T cells, Ly6C^**+**^Ly6G^**+**^CD4^**+**^ T cells expressed higher levels of *Mmp9*, *Ccl3*, *Ccl4*, *Cxcl2*, *Cxcl3*, *Nlrp3* and *Il1a* (Fig. 5A). Further, KEGG enrichment and the GSEA analysis showed that the differentially expressed genes involved in cytokine-cytokine receptor interaction were highly expressed in Ly6C^**+**^Ly6G^**+**^CD4^**+**^ T cells (Fig. 5B, C), indicating the Ly6C^**+**^Ly6G^**+**^CD4^**+**^ T cells were in an activated status.

Next, CD4^+^ T cells were divided into Ly6C^**+**^Ly6G^**+**^CD4^**+**^ T cells, Ly6C^**+**^Ly6G^**-**^CD4^**+**^ T cells, Ly6C^**-**^Ly6G^**+**^CD4^**+**^ T cells, and Ly6C^**-**^Ly6G^**-**^CD4^**+**^ T cells, based on the differential expression of Ly6C and Ly6G. The capacities of the four subsets to secrete IL-10 (Fig. 5D, E), IL-21 (Fig. 5F, G), granzyme B (Fig. 5H, I), and perforin (Fig. 5J, K) were detected by flow cytometry. It was found that Ly6C^+^Ly6G^+^CD4^+^ T cells secreted significantly higher IL-10, IL-21, granzyme B, and perforin than that of the other three clusters (Fig. 5D–K). After performing a similar analysis on CD8^+^ T cells, Ly6C^+^Ly6G^+^CD8^+^ T cells also had stronger IL-10 and IL-21 secretion ability than the others analyzed, while the ability of Ly6C^+^Ly6G^+^CD8^+^ T cells to secrete perforin and granzyme B was not the highest among the CD8^+^ T cell populations (Supplementary Fig. 4). Moreover, Ly6C^+^Ly6G^+^CD4^+^ T cells produced a higher amount of IL-21 than Ly6C^+^Ly6G^+^CD8^+^ T (Supplementary Fig. 5A). Interestingly, the production of granzyme B and perforin was greater in these CD4^+^ T than CD8^+^ T cells. There was no significant difference in IL-10 production between these two clusters (Supplementary Fig. 5B–D). Moreover, the IL-21^+^Granzyme B^+^ cells among Ly6C^+^Ly6G^+^CD4^+^ T cells were CXCR5 positive cells (Fig. 5L), indicating Ly6C^+^Ly6G^+^CD4^+^ T cells included cytotoxic T follicular helper (Tfh) cells. Thus, Ly6C^+^Ly6G^+^CD4^+^ T cells possess the ability to strongly secrete IL-10, IL-21, perforin, and granzyme B.

### Ly6C^+^Ly6G^+^CD4^+^ T cells promote macrophages polarization from M2 to M1 phenotype post-APP infection

**Fig. 6:**
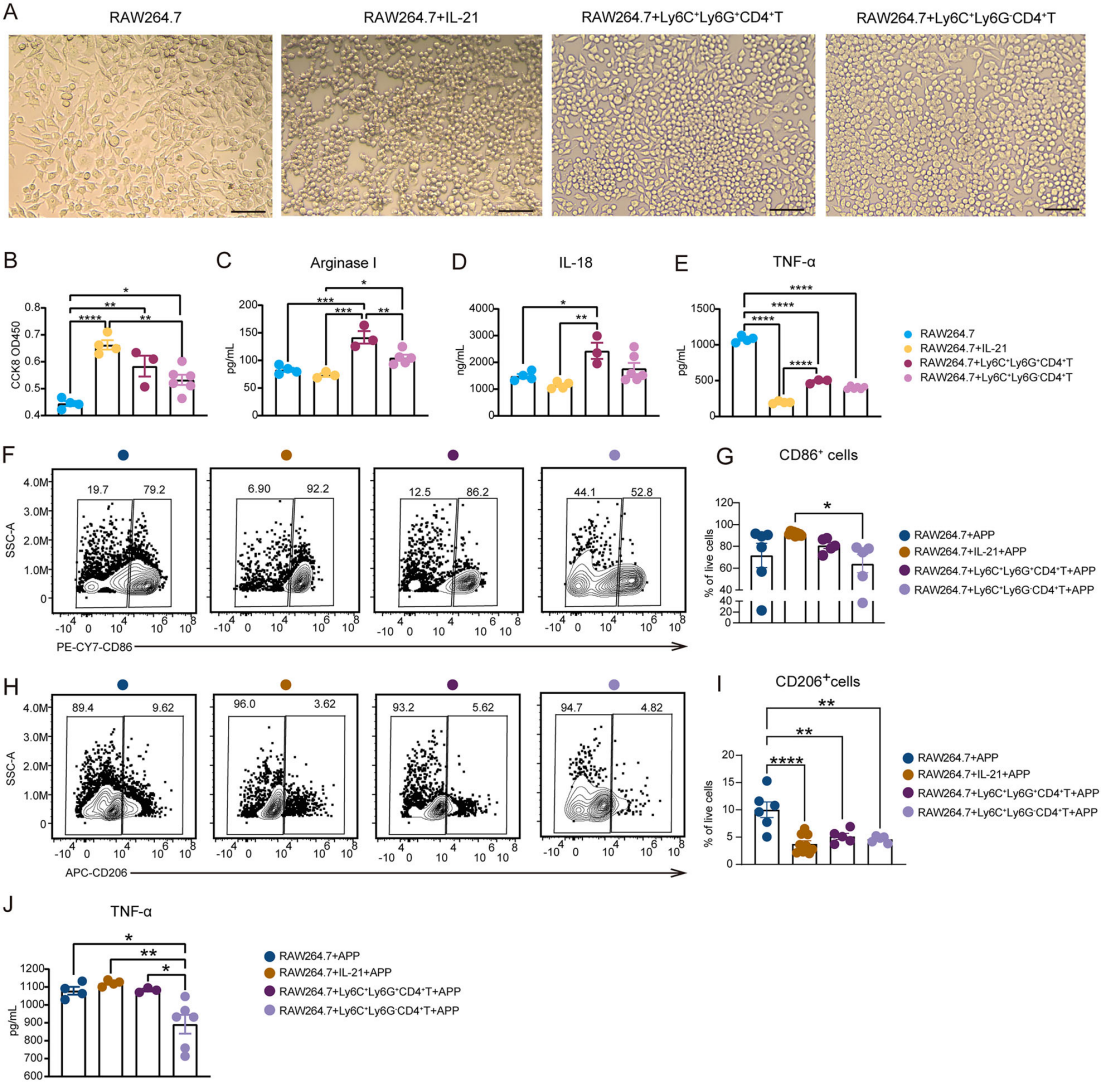
Ly6C^+^Ly6G^+^CD4^+^ T cells promote macrophage polarization to M1 state after APP infection. **A**–**E** RAW264.7 macrophages were co-cultured with IL-21, Ly6C^+^Ly6G^+^CD4^+^ T cells, and Ly6C^+^Ly6G^-^CD4^+^ T cells for 84 h. **A** The microscopic morphology of each group of cells (under 40x microscope, scale bar=100 μm). **B** CCK8 experiments detect the proliferation of RAW264.7 macrophages. **C**–**E** ELISA assays detect the content of (**C**) Arginase I, (**D**) IL-18, and (**E**) TNF-α in the cell culture supernatant. **F**–**J** After co-culture, RAW264.7 macrophages were stimulated with APP (MOI = 5) for 30 min, the gentamicin was then added to remove extracellular bacteria. After that, the cells were cultured for 6 h to detect the macrophage polarization. **F**–**I** The representative gating strategies and percentage of CD86^+^ and CD206^+^ cells detected by flow cytometry. **J** The content of TNF-α in the culture supernatant. *p < 0.05; **p < 0.01; ***p < 0.001; ****p < 0.0001, using Mann-Whitney U test. Error bars show means ± SEM.

Further research is required to determine whether Ly6C^+^Ly6G^+^CD4^+^ T cells aided the innate immune response against APP infection. Ly6C^+^Ly6G^+^CD4^+^ T cells from the lungs of WT mice infected with APP as well as Ly6C^+^Ly6G^-^CD4^+^ T cells and recombinant protein IL-21 were co-cultured with RAW264.7 macrophages, respectively. The findings revealed that single-cultured macrophages had a spindle shape, but the other three cell groups had rounded or oval shapes (Fig. 6A), indicating that macrophages had been effectively activated. However, both IL-21 supplementation and co-culture with Ly6C^+^CD4^+^ T cells, especially Ly6C^+^Ly6G^+^CD4^+^ T, considerably augmented the growth of macrophages (Fig. 6B). Ly6C^+^Ly6G^+^CD4^+^ T cells dramatically enhanced the levels of M2-polarization associated molecules Arginase I and IL-18 in the co-culture supernatant microenvironment, whereas IL-21 alone had no discernible effect on the secretion of Arginase I and IL-18 (Fig. 6C, D), indicating that this function of Ly6C^+^Ly6G^+^CD4^+^ T cells was not entirely dependent on IL-21. Additionally, compared to the RAW264.7 group, the co-culture with Ly6C^+^Ly6G^+^CD4^+^ T cells significantly reduced the production of TNF-α in the co-culture cell supernatant (Fig. 6E), achieving a similar outcome to the IL-21 addition group (Fig. 6E). Our data indicate that Ly6C^+^Ly6G^+^CD4^+^ T cells kept the macrophages in the M2 state without infection.

Then, we assessed the effects of Ly6C^+^Ly6G^+^CD4^+^ T cells on RAW264.7 macrophages after APP infection. The fractions of M1-type macrophages indicated by CD86 expression were increased in the IL-21 supplementation group and Ly6C^+^Ly6G^+^CD4^+^ T cells group post-infection compared to the RAW264 cell alone group (Fig. 6F, G), indicating this function may partly depend on the IL-21/IL-21 signaling axis. Meanwhile, the three addition groups drastically reduced the expression of CD206 on the surface of macrophages, preventing them from polarizing into M2 type (Fig. 6H, I). In contrast to Ly6C^+^Ly6G^-^CD4^+^ T cells group, the suppression of TNF-α secretion in IL-21 or Ly6C^+^Ly6G^+^CD4^+^ T group was reduced after APP infection. Furthermore, there was no discernible difference between RAW264.7 macrophages alone, Ly6C^+^Ly6G^+^CD4^+^ T cells co-culture, and the IL-21 addition group in terms of TNF-α levels in the cell culture supernatant, suggesting that IL-21 is potentially responsible for inducing TNF-α production after APP infection (Fig. 6J). Thus, Ly6C^+^Ly6G^+^CD4^+^ T cells promote macrophages polarization from M2 to M1 phenotype post-APP infection.

### Ly6C^+^Ly6G^+^CD4^+^ T cells boost macrophages to phagocytose and kill bacteria by METs or ROS

**Fig. 7:**
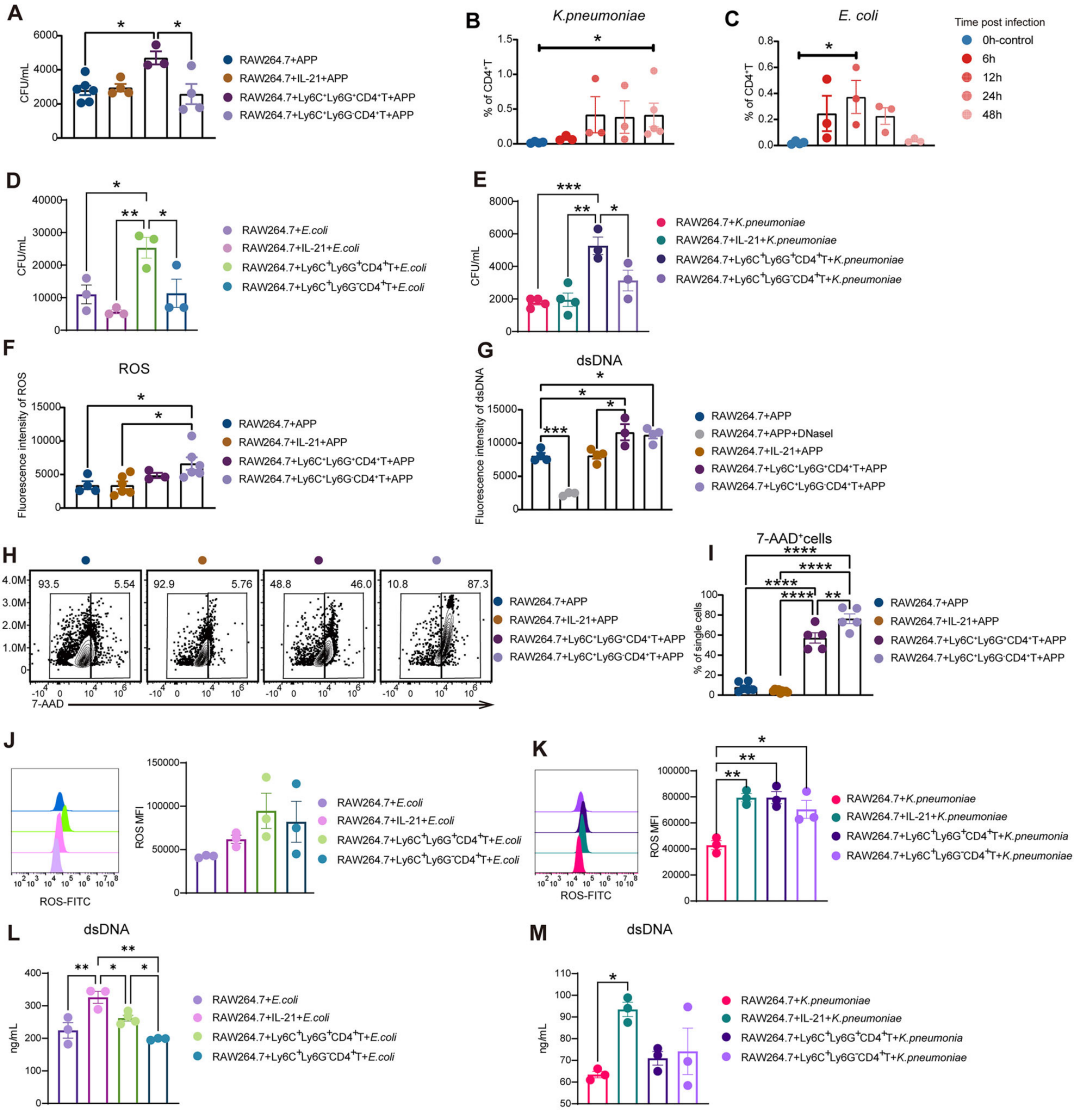
Ly6C^+^Ly6G^+^CD4^+^ T cells increase the phagocytosis and bactericidal activity of macrophages. **A** RAW264.7 macrophages were co-cultured with IL-21, Ly6C^+^Ly6G^+^CD4^+^ T cells, and Ly6C^+^Ly6G^-^CD4^+^ T cells for 84 h. After co-culture the RAW264.7 macrophages were stimulated with APP (MOI = 10) for 30 min, APP content in macrophages were analyzed. **B**, **C** The proportion of Ly6C^+^Ly6G^+^CD4^+^T cells in CD4^+^ T cell population in the lung of ICR mice infected with *K. pneumoniae* and *E. coli* at 0, (without infection), 6, 12, 24, and 48 h, respectively. **D**, **E** RAW264.7 macrophages were co-cultured with IL-21, Ly6C^+^Ly6G^+^CD4^+^ T cells, and Ly6C^+^Ly6G^-^CD4^+^ T cells for 84 h. RAW264.7 macrophages were stimulated with *E.coli* and *K.pneumoniae* (MOI = 10) for 30 min, respectively, the bacterial content in macrophages were analyzed. With the same treatment in (**A**), **F** the ability of macrophages to produce ROS, and **G** the content of dsDNA in the cell supernatant were detected. **H**, **I** Representative gating strategies of 7-AAD^+^ cells and the quantification of 7-AAD^+^ cells. **J**, **K** The ability of macrophages to produce ROS detected by flow cytometry, and **L**, **M** the content of dsDNA in the cell supernatant of macrophages treated as (**D**) and (**E**).

We next assessed the effects of Ly6C^+^Ly6G^+^CD4^+^ T cells on the phagocytosis and bactericidal activity of macrophages, which are essential for anti-infection immunity. Compared to Ly6C^+^Ly6G^-^CD4^+^ T cell co-culture group and the macrophage alone group, the phagocytic bacterial number of macrophages in Ly6C^+^Ly6G^+^CD4^+^ T cell co-culture group was significantly higher, while IL-21 addition alone did not improve the phagocytosis of macrophages (Fig. 7A). This indicated that the augmented phagocytosis in Ly6C^+^Ly6G^+^CD4^+^ T cell group may not depend on the IL-21. Interestingly, the Ly6C^+^Ly6G^+^CD4^+^ T cells were also increased in the mouse lungs post-*Klebsiella pneumoniae*, or *Escherichia coli* infection (Fig. 7B, C, Supplementary Fig. 6). Also, enhanced phagocytosis in Ly6C^+^Ly6G^+^CD4^+^ T cell co-culture group were obtained in *E. coli* or *K. pneumoniae* infection (Fig. 7D, E).

ROS are fundamental for macrophages to eliminate invasive bacteria. However, there were no statistical differences between the Ly6C^+^Ly6G^+^CD4^+^ T cell co-culture group and the macrophages alone group post-APP infection (Fig. 7F). Interestingly, the Ly6C^+^Ly6G^+^CD4^+^ T cell co-culture group considerably outperformed the macrophage alone and IL-21 addition groups in terms of their ability to generate dsDNA (Fig. 7G). Meanwhile, compared to macrophage alone and the IL-21 supplementation groups, Ly6C^+^Ly6G^+^CD4^+^ T cell or Ly6C^+^Ly6G^-^CD4^+^ T cell co-culture with macrophages induced more macrophage death (Fig. 7H, I). Based on the development of macrophage extracellular traps (METs), these data indicate that Ly6C^+^Ly6G^+^CD4^+^ T cells could increase the bactericidal activity of macrophages by METs. After *E. coli* or *K. pneumoniae* infection, both IL-21 addition and T cell co-culture with macrophages could enhance the ROS release from macrophages, especially in the *K. pneumoniae* infection (Fig. 7J, K), indicating ROS generation of macrophages in the above two types of infection may depend on the IL-21. In contrast to the APP infection, compared to the macrophage infection alone group, the IL-21 addition but not the T cell co-culture group promoted macrophages to produce significantly higher amounts of dsDNA in *E. coli* or *K. pneumoniae* infections. Altogether, our data indicate that Ly6C^+^Ly6G^+^CD4^+^ T cells could boost macrophages to phagocytose and kill bacteria mediated by METs or ROS.

### CD14^+^CD4^+^ T cells promote porcine alveolar macrophages to kill bacteria by METs or ROS

**Fig. 8:**
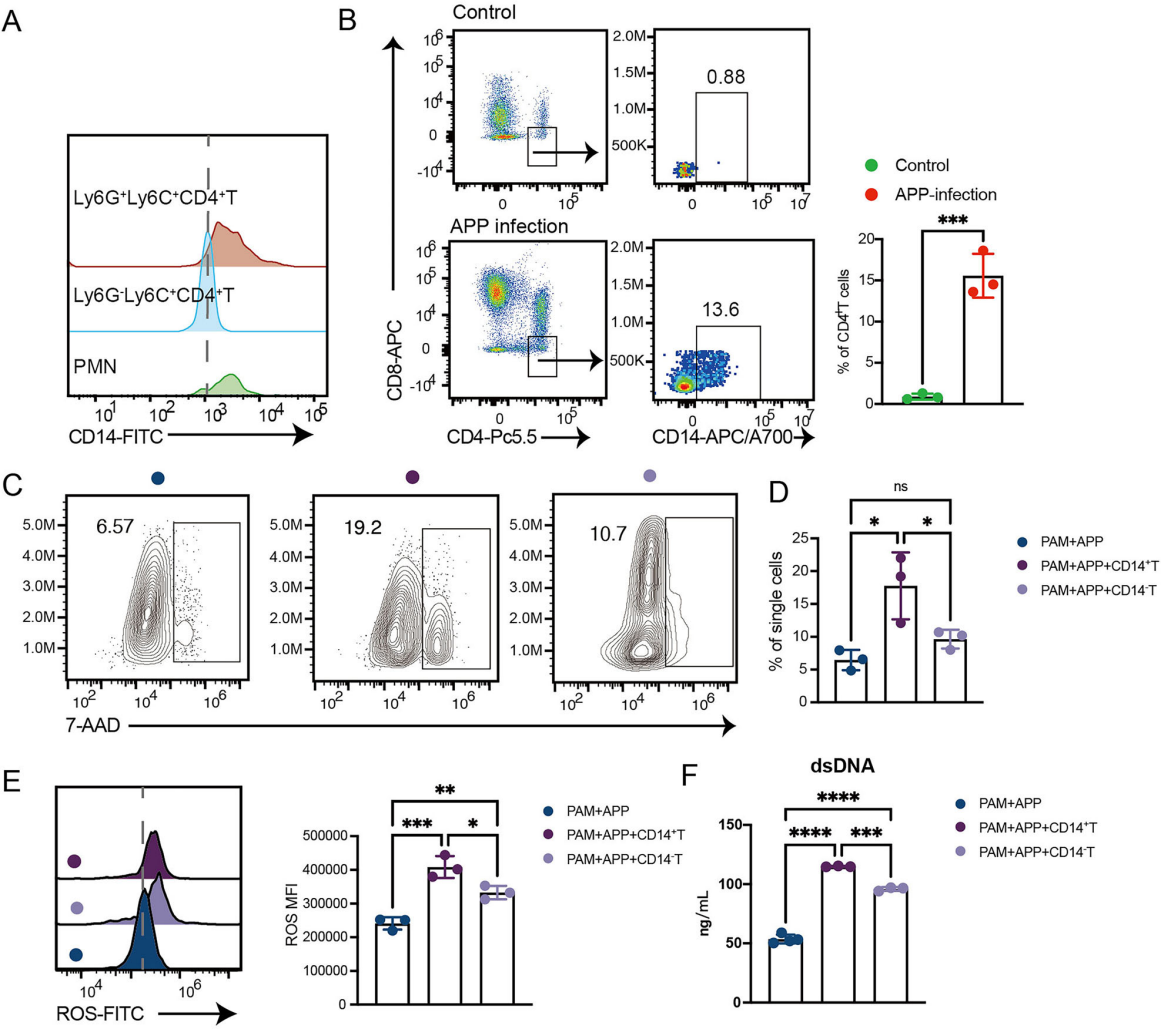
CD14^+^CD4^+^ T cells increase the bactericidal activity of porcine alveolar macrophages. **A** Mean fluorescence intensity (MFI) of CD14 expression on Ly6C^+^Ly6G^+^CD4^+^ T cells, Ly6C^+^Ly6G^-^CD4^+^ T cells and PMN. **B** Flow cytometry was used to detect the proportion of CD14^+^CD4^+^T cells in CD4^+^T cells in porcine lung tissues after APP infection. **C**, **D** Representative gating strategies of 7-AAD^+^ cells and the quantification of 7-AAD^+^ cells. **E** The ability of macrophages to produce ROS detected by flow cytometry, and **F** the content of dsDNA in the cell supernatant of PAMs treated as (**E**).

Finally, we investigated whether Ly6C^+^Ly6G^+^CD4^+^T cells in pigs have immune functions similar to mice. Due to the lack of Ly6G and Ly6C molecules in pigs, we have looked for markers that can replace Ly6C and Ly6G. Interestingly, the expression of CD14 in Ly6C^+^Ly6G^+^CD4^+^T cells was significantly higher than that in Ly6C^+^Ly6G^-^CD4^+^T cells (Fig. 8A). Moreover, the proportion of CD14^+^CD4^+^T cells increased in pig lungs after APP infection, and there were almost no CD14^+^CD4^+^T cells in the control group (Fig. 8B), which is consistent with the results in mice. Thus, we used CD14^+^CD4^+^T cells in place of Ly6C^+^Ly6G^+^CD4^+^T cells on pigs. Next, we explored whether CD14^+^CD4^+^T cells can also promote the killing of APP by porcine alveolar macrophages (PAMs). We co-cultured CD14^+^CD4^+^T cells with PAMs. Compared to the PAMs alone group, CD14^+^CD4^+^T cells co-culture with PAMs induced more macrophage death (Fig. 8C). Furthermore, there was no discernible difference between the CD14^-^CD4^+^T cell co-culture and the PAMs alone group (Fig. 8D). Compared with the PAMs alone, both CD14^+^CD4^+^T cells and CD14^-^CD4^+^T cells could promote the production of ROS and higher levels of dsDNA in PAMs, and the promoting effect was more significant in CD14^+^CD4^+^T cells (Fig. 8E, F). In summary, these data indicated that we had found CD14^+^CD4^+^T cells in pigs’ lungs with similar functions to mouse Ly6C^+^Ly6G^+^CD4^+^T cells post-APP infection, which could promote PAMs to kill APP mediated by METs or ROS.

## Discussion

To our knowledge, little is known about the role of IL-21 in mediating resistance to bacterial lung infection. In this study, we found that IL-21 had a protective effect on the induction of the early innate immune response in mouse lung post-APP infection. The heterogeneity of lung immune cells was further analyzed by high-throughput mass cytometry, and it was found that IL-21R deficiency inhibited the differentiation and activation of key innate immune cells such as neutrophils and macrophages and promoted the defective CD3^low^ T cells accumulation in the lung in the early stage of infection. For the first time, IL-21-induced Ly6C^+^Ly6G^+^ T cell subsets, which had not been reported before, were found to confer resistance to APP infection in mice. Compared with other T cell clusters, this subset had a higher secretion capacity of IL-10, IL-21, perforin, and granzyme B. What’s more, the expression of CXCR5 on these cells indicated that the subset may be cytotoxic T follicular helper (Tfh) cells. The ability to secrete IL-21 directly promoted macrophage proliferation. Ly6C^+^Ly6G^+^CD4^+^ T cells kept macrophages polarization in the M2 state without infection and promoted macrophages to M1 phenotype post-APP infection to enhance the anti-infection effect. It also promoted phagocytic and dsDNA-mediated killing function of macrophages post-APP, infection, but it was not directly regulated by IL-21. Interestingly, this novel subset was also identified in the lung with *E. coli* or *K. pneumoniae* infection and could assist the macrophages against the infections. This study demonstrated heterogeneity of immune cell response to bacterial-induced lung infection and revealed the mechanism by which IL-21 protects the host against bacterial infection, broadening the horizons of T cell function, and providing the novel targets for drug and vaccine development for APP even other infection-associated pneumonia.

Consistent with previous studies [28, 29], we identified neutrophils, inflammatory monocytes, M1-type macrophages, interstitial macrophages, M1-type AM, M2-type AM, and dendritic cells as reported in the mouse lung. Furthermore, our study revealed unprecedented heterogeneity in the immune compartment in the mouse lung. For example, nine distinct phenotypic neutrophil clusters were identified in the lung. IL-21 was found to up-regulate the expression of CD11b and CD16 in IL-21R^+^ neutrophils from human peripheral blood [30]. Our results showed that most neutrophils in the lungs of IL-21R^−^^/−^ mice were in an inactivated state (low expression of CD11b and CD45), highlighting the function of IL-21 in promoting the activation of neutrophils. Beyond that, MHC-Ⅱ positive neutrophils were found, suggesting that they may be neutrophil-DC cells. However, unlike previous studies of neutrophils-DCs [31], these cells did not express CD11c (the DC cell surface marker), further highlighting the unique characteristics of neutrophils in the lung. Our studies on cell heterogeneity have challenged the traditional understanding of cellular phenotypes and enriched our understanding of cells, however, it is necessary to further explore the functions of these new cell clusters in physiological and pathological states.

In addition, monocytes and macrophages also displayed strong heterogeneity. M1-type macrophages were significantly reduced due to IL-21R deficiency after APP infection, suggesting that IL-21 may affect the recruitment or differentiation of M1-type macrophages. Meanwhile, macrophages differentially expressed SiglecF. In bleomycin-induced lung injury, SiglecF low expressed AM were the intermediate phenotype of the redifferentiation of IM into AM [28]. In our study, M2-type AM with low expression of SiglecF were significantly reduced in IL-21R^−^^/−^ mice, suggesting that IL-21 was involved in the regulation of differentiation processes of AM. Although our study was the first to systematically reveal the pulmonary immune response after APP infection, the lack of chemotaxis, differentiation, cytokines, and other marker proteins of myeloid cells limited the in-depth study of these cells and will form the basis of future studies.

In addition to complementing the heterogeneity of myeloid cells affected by IL-21 in the innate immune response, IL-21/IL-21R-induced T cell cluster expressing both Ly6C and Ly6G on its surface was also identified for the first time. Ly6C had been reported to be expressed on the surface of CD4^+^ T cells and CD8^+^ T cells previously. Ly6C^+^CD4^+^ T cells were mainly effector T cells with a strong ability for cytokine secretion [32], while Ly6C^+^CD8^+^ T cells, as an activated T cell phenotype, played an important role in promoting acute lung injury [33]. Ly6G, as a marker mainly expressed on the surface of neutrophils, had not been found on the surface of T cells in previous studies [34]. In the current study, we found that Ly6C and Ly6G double-positive CD4^+^ T cells and CD8^+^ T cells had strong secretion capacities of IL-10, IL-21, perforin, and granzyme B, while Ly6C^+^Ly6G^+^CD4^+^ T cells had a stronger ability than Ly6C^+^Ly6G^+^CD8^+^ T cells.

IL-10 is a typical anti-inflammatory cytokine. However, Brockmann., et al found that IL-10-producing CD4^+^ T cells are phenotypically and functionally heterogeneous, and a subpopulation of IL-10-producing CD4^+^ T cells were unexpectedly identified as pro-inflammatory cells [35]. Accordingly, the function of the IL-10-producing Ly6C^+^Ly6G^+^CD4^+^ T cells discovered here needs to be elucidated in the future. While IL-21 can directly promote the polarization of macrophages to M2-type macrophages [23, 36], inhibit the production of pro-inflammatory cytokines by dendritic cells [37], induce the apoptosis of dendritic cells [38], and promote the maturation, differentiation, and secretion of pro-inflammatory cytokines of natural killer cells and T cells [39, 40]. To the extent of the findings before, our data proved that IL-21 played a direct role in promoting macrophage proliferation, whereas the direct effect of IL-21 on phagocytosis was not obvious. Moreover, the increased number of Ly6C^+^Ly6G^+^CD4^+^ T was induced by IL-21/IL-21R post-infection, suggesting that IL-21/IL-21R signaling may play an anti-infection role through regulating the intercellular network. Also, the regulation of macrophage function by the novel T cell cluster further indicated that adaptive immune cells may participate in anti-infection immunity at the early stage of infection. The way of IL-21/IL-21R signaling regulated the production of Ly6C^+^Ly6G^+^CD4^+^ T cells, as well as the effect of this novel cluster on different immune cells and its regulatory mechanism needs to be further elucidated.

In the aspect of perforin and granzyme B, it breaks our inherent cognitive that CD8^+^ T cells have stronger perforin and granzyme B release capacity than CD4^+^ T cells. It has been reported that CD4^+^ T cells, such as memory CD4^+^ T cells [41] and cytotoxic CD4^+^ T cells [42, 43] can secrete granzyme B and perforin. Moreover, CD4^+^ T cells expressing granzyme B and perforin are often associated with cytotoxic effects [44], such as mediating cell death [45, 46], histopathological injury [47], or effectively control viral infections [42, 47]. However, granzyme B^+^ CD4^+^ T cells are also involved in inflammatory responses and extracellular matrix remodeling [33], even prevents aberrant IL-17 production in CD4^+^ T cells [48]. Therefore, Ly6C^+^Ly6G^+^CD4^+^ T cells may be a subset of cytotoxic CD4^+^ T cells with a memory phenotype, which may have cell killing and apoptosis-inducing properties. If perforin and granzyme B secreted by Ly6C^+^Ly6G^+^CD4^+^ T cells mediated the METs function of macrophages it still needs to be investigated. At the same time, it is not ruled out that Ly6C^+^Ly6G^+^CD4^+^ T cells may also be involved in regulating the differentiation or activation of T cells. It is noteworthy that the cells were universal in respiratory diseases induced by a variety of pathogens, further emphasizing the importance of investigating this cell subset in bacterial pneumonia.

In this study, CD4^+^ T cells identified in the mice lung were found to have low expression of CD3e, TCRβ, or both, which was more abundant in the IL-21R^−^^/−^ mice. CD3epsilon (CD3e) is often used as an antibody epitope to recognize CD3 molecules on T cells because it can be expressed stably and continuously on the surface of T cells after maturation and can activate T cells by transforming TCR recognition of antigen into intracellular signal cascade reaction [49]. However, CD3e expression in T cells was downregulated in hosts with cancer [50] or infection with immunodeficiency virus [51, 52], resulting in T cell dysfunction and impaired cellular immune response. Lung transcriptome sequencing results of APP piglet infection model established in the early stage revealed that the expression of lung lymphocyte activation-related genes was significantly down-regulated at the early stage of APP infection (6 h and 12 h) [22]. Therefore, CD3e^low^CD4^+^ T cells was caused mainly by IL-21/IL-21R deficiency, which could impair lung immune response in the early stage of APP infection. In addition, several microbial pathogens evade or alter T cell-mediated adaptive immunity by avoiding T cell recognition or by interfering with T cell priming. For example, *S. Typhimurium* secreted a factor that reduced TCRβ expression upon contact with T cells, thereby inhibiting T cell priming [53]. APP has a strong ability to secrete virulence factors, thus it cannot be ruled out that APP reduces the expression of TCRβ on the surface of T cells after exposure to lung T cells.

T cells are plastic and can form functional subsets of immune cells in different immune microenvironments. Due to the low proportion of Ly6C^+^Ly6G^+^CD4^+^ T cells in the lung, we first tried to sort out these cells in the lung and then amplify them in vitro using different cytokines. Although the T cells with Ly6C^+^Ly6G^+^CD4^+^ phenotype were successfully expanded, the generated cells were without the ability to produce IL-10 and IL-21 (data not shown). Secondly, we focused on the spleens, an organ rich in T cells, and found that the ability of CD4^+^Ly6C^+^Ly6G^+^ T cells to secrete IL-10 in the spleen was different from that in the lung, indicating the key phenotype of cells was the same, however, the cell function was different. Therefore, we can only isolate this subset from the lung cells of APP-infected mice for further functional study in the present study. In the subsequent studies, the cytokine receptors on the surface of Ly6C^+^Ly6G^+^CD4^+^ T cells will be screened, and the combination of cytokines would be used to gradually screen out the best conditions for in vitro amplification and culture of these T cells.

In conclusion, our study was the first to reveal that IL-21/IL-21R signaling could promote the activation and maturation of the innate immune cells in the lung and had a role in resistance to bacterial infection. In addition, we found IL-21R-induced T cells expressing Ly6C and Ly6G on the cell surface, among which CD4^+^ T cells could shape the macrophage polarization in different states with or without infection, and promote macrophage proliferation, phagocytosis, and METs/ROS-mediated sterilization against bacterial infection. This provides the new targets for the development of drugs and vaccines for bacterial pneumonia and enriches the knowledge of T cells. It should be noted that while mice provide a valuable and tractable model for mechanistic studies, the immune responses may differ from those in pigs, the natural host of APP, and this limitation should be considered when translating findings.

## Materials and methods

### Mice and bacterial strains

C57BL/6 mice were purchased from Liaoning Changsheng Biotechnology Co., Ltd. (permit number: 210726211100519064). IL-21R^−^^/−^ mice (C57BL/6 background) were gifted from Prof. Y. Zhinan at Jinan University. All mice were housed under specific pathogen-free conditions at Jilin University, and efforts were made to minimize animal suffering.APP serotype 5 reference strain L20 (APP 5b L20) was obtained from the Shanghai Entry-Exit Inspection and Quarantine Bureau (Shanghai, China) and grown in brain-heart infusion (BHI) broth supplemented with 15 μg/mL nicotinamide adenine dinucleotide (NAD) at 37 °C. *Klebsiella pneumoniae* kp36 and *Escherichia coli* are preserved in our laboratory, cultured in Luria-Bertani (LB) medium at 37 °C. For infection of mice, an isolated colony was transferred to 3 mL of brain-heart infusion (BHI) medium and incubated for 6 h at 37 °C with 180 rpm agitation. The bacteria were centrifuged at 3500×g and washed three times with phosphate-buffered saline (PBS).

### Mouse survival rate

Six IL-21R^−^^/−^ and six WT mice weighing 18-20 g were used to instill 30 μL of 3.2 ×106 CFU APP bacterial suspension after anesthesia by inhalation of 2.5% isoflurane. The survival rate of WT and IL-21^−^^/−^ mice were observed within 72 h post APP infection. And the mice that lost 20% of their initial body weight were considered to have reached the experimental endpoints and were humanely euthanized by cervical dislocation.

### Experimental infection

WT and IL-21R^−^^/−^ female mice weighing 18-20 g were randomly divided into 4 groups: WT+saline, IL-21R^−^^/−^+saline, WT + APP, and IL-21R^−^^/−^ + APP. Due to the high susceptibility of IL-21R^−^^/−^ mice to APP, a low dose of APP (1×10^6^ CFU) was used to study the characteristics of immune response in the lung. WT, and IL-21R^−^^/−^ mice in the APP-infected group were injected with APP in a 30 μL bacterial solution intranasally, while the mice in the control group were injected with 30 μL sterile saline. After 12 h post infection, the above 4 groups of mice were euthanized, and their body weight was recorded. After separation, the lungs were weighed and photographed. Subsequently, the same part of the lung tissue of each group of mice was fixed in 4% formaldehyde solution, and H&E sections were prepared. The remaining lung tissue was used to prepare a single cell suspension.

### In vivo addition of IL-21

C57BL/6 mice were randomly divided into two groups (6 mice/group), including the IL-21 administration group and the control group. Mice in these two groups were anesthetized with 1% pentobarbital sodium 24 h before infection. The IL-21 administration group was given 2 μg of IL-21 (dissolved with 20 μL of 0.1%BSA), and the control group was given 0.1% BSA of the same volume. Mice infected with 1×10^6^ CFU APP for 12 h were recorded the clinical signs, then euthanized. The lungs were photographed, weighed, and lung bacterial load was calculated by plate counting.

### Real-time PCR for quantification of cytokine mRNA

Total RNA was extracted from the lung tissues as described previously [54]. RNA was reverse transcribed into cDNA using a reverse transcription kit (Takara, Tokyo, Japan). The PCR reaction included 10 μL SYBR Green (Takara, Tokyo, Japan), 7.4 μL water, 1.6 μL primers, and 1 μL template, and the expression levels of *IL-21, IL-10, TGF-β*, and *IFN-γ* were determined. Primers used in this study are listed in Supplementary table 1.

### Lung cell isolation

Lungs were removed from mice in the different groups, minced, and incubated in 1 mL tissue digestive solution containing 0.3 mg/mL collagenase IV (Sigma-Aldrich, St.145 Louis, MO, USA), 25 U/mL DNAse I (Solarbio, Beijing, China) and 5% Fetal Bovine Serum (FBS) in RPMI (Biological Industries, Kibbutz Beit Haemek, Israel) for 30 min at 37 °C. After digestion, a syringe was used to lyse the cells into homogenates, and erythrocytes were lysed by the addition of red blood lysis buffer (Solarbio, Beijing, China), followed by two washes with FACS buffer (PBS supplemented with 0.5% FBS), before resuspension of cells in FACS buffer.

### Mass cytometry and antibody staining

A panel of 25 antibodies designed to distinguish a broad range of immune cells was used. Antibodies were purchased from Fluidigm (South San Francisco, CA, United States), Biolegend (San Diego, CA, United States), or Abcam (Cambridge, MA, United States). The purified antibodies lacking carrier protein were conjugated with metal reporters using the MaxPar X8 antibody labeling Kit (Fludigm Science) according to the manufacturer’s instructions. Antibodies and reporter isotopes used for mass cytometry are listed in Supplementary table 2.

Briefly, cell samples were washed and incubated with 0.5 mL 2 μM Cell-ID Cisplatin to identify dead cells, and then stained with cell surface antibodies for 30 min on ice. The antibody-labeled samples were washed and incubated in 0.125 mM intercalator-Ir (Fluidigm, South San Francisco, CA, United States) diluted in PBS containing 2% formaldehyde to identify all cells and stored at 4°C until mass cytometry analysis. The samples were washed with deionized water and then resuspended at a concentration of 1×10^6^ cells/mL in deionized water containing EQ Four Element Beads (1:20 dilution) (Fluidigm, South San Francisco, CA, United States). The samples were examined by Helios Mass Cytometer (Fluidigm, South San Francisco, CA, United States).

### Mass cytometry data analysis

Data from single, live, and CD45^+^ cells were gated individually using FlowJo software (version 10.5.3) as shown in Figure S7 and then were sample-tagged and hyperbolic-arcsinh-transformed with a cofactor of 5 using Cytosplore^+H-SNE^ software [55]. The hierarchical stochastic neighbor embedding (H-SNE) analysis was carried out with default setting (Perplexity:30; iteration:1000) in Cytosplore. All the H-SNE, t-SNE plots and Gaussian Mean-shift clustering-derived cell clusters were generated using Cytosplore. Each cluster contains at least 100 cells. Hierarchical clustering of the phenotype heatmap was generated with Euclidean correlation and average linkage clustering in Matlab 2015. The cell frequency bar graphs were created by GraphPad Prism 9 software.

### Flow cytometry staining

Lung mononuclear cells were aseptically collected from the different groups of mice and the cells were incubated with antibodies for 30 min on ice in the dark for surface marker analysis. For intracellular cytokine staining, lung single cells were stimulated with PMA (50 ng/mL) and ionomycin (1 mg/mL) for 6 h at 37°C, and 1x brefeldin A and monensin solution (BioLegend) were added for the final 4 h. Cultured cells were washed twice using FACS buffer, and stained with surface markers The cells were then fixed, permeabilized with Cyto-Fast™ Fix/Perm Buffer Set (BioLegend) and incubated with intracellular antibodies for 30 min at 4°C. Raw data were collected on a FACS Canto II flow cytometer (BD Biosciences) and analyzed using Flow Jo 10.0 software. The details of the antibodies we used for flow cytometry are listed in Supplementary table 3.

### Transcriptome analysis of Ly6C^+^Ly6G^+^CD4^+^T and Ly6C^+^Ly6G^-^CD4^+^T cells

Briefly, RNA was extracted by RNeasy Kits (Qiagen, Catalog number. 75144) and treated with DNase I (Thermo Fisher Scientific) according to the standard protocols. RNA quality was accessed by Bioanalyzer and those RIN higher than 7 were used to construct the RNA-seq library with Smart-seq2 method. For Smart-seq2 library preparation, 200 pg of starting total RNA was used in template-switching reverse transcription reaction. After pre-amplification and AMPure XP beads purification, cDNA was subject to Tn5 transposase-based library preparation. The final library quality assessment was performed using BioAnalyzer and Qubit. All libraries were sequenced on Illumina NovaSeq (150 bp, paired-end). After quality control, sequence data were processed with STAR to generate read alignments with hg19. Raw read counts for annotated genes were obtained with feature counts with default settings, normalized and analyzed using DEseq2. Transcriptome sequencing and data analysis were performed by Tianhao Biological Co., Ltd.

### Cell sorting, co-culture with RAW264.7 cells, morphological observation, and proliferation detection of RAW264.7 cells

T cells derived from the lungs were enriched using the EasySep™ Mouse CD90.2 Positive Selection Kit II (stem cell), and enriched T cells were then labeled with CD4-FITC, Ly6C-APC, and Ly6G-PerCP-eFluor 710 to enable sorting of Ly6C^+^Ly6G^+^CD4^+^ T cells and Ly6C^+^Ly6G^-^CD4^+^ T cells by BD Influx (BD Biosciences). 2,000 Ly6C^+^Ly6G^+^CD4^+^ T or Ly6C^+^Ly6G^-^CD4^+^ T cells were co-cultured with 4,000 RAW264.7 cells in each well of a 96-well plate for 84 h in RPMI medium containing 10% FBS, 2 mM L-glutamine, 10 mM HEPES buffer, 1 mM sodium pyruvate, 100 μM MEM Non-essential amino acids, β-mercaptoethanol, 1x penicillin-streptomycin solution, rIL-2 (100 U/mL), CD3 (0.5 μg/mL), and CD28 (0.5 μg/mL). Supernatants were collected to determine the cytokine levels by ELISA according to the instructions of individual kits. Cell morphology was observed and photographed using an inverted microscope (Olympus IX73). Due to the adherent properties of RAW264.7 cells and the suspension properties of T cells, the co-cultured cells were washed three times with sterile PBS to remove the suspended T cells. The proliferation assays of the RAW264.7 cells after co-culture in each group were then performed using a CCK8 Assay Kit (APExBIO), according to the instructions.

### Detection of macrophages polarization and death by flow cytometry

To determine the effect of Ly6C^+^Ly6G^+^CD4^+^ T cells on macrophage polarization after APP infection, the co-cultured cells were first washed three times with sterile PBS to remove the suspended T cells as mentioned above. The co-cultured RAW264.7 cells were then collected, labeled with 7-AAD (BioLegend), CD86-PE/Cy7 (BioLegend) and CD206-APC (BioLegend) as described in flow cytometry staining section above, and detected by flow cytometry.

### Detection of bacteria inside the RAW264.7 cells after co-culture

To determine the effect of Ly6C^+^Ly6G^+^CD4^+^ T cells on macrophage function, the numbers of APP, *K. peumoniae* and *E. coli* inside the RAW264.7 cells were determined after 1 h of coculture (MOI = 10). To enumerate the bacteria present in RAW264.7 cells in each group, cells were washed three times with sterile PBS buffer, and 0.1 mL of RPMI containing 100 μg/mL of gentamicin was added to each well and incubated at 37 °C with 5% CO2 for 10 min, then the cells were washed three times with sterile PBS buffer. Finally, RAW264.7 cells were lysed with 0.1 mL of RPMI medium containing 1% (v/v) Triton X-100 (Sigma, USA) for 10 min and the RAW264.7 cell lysates (0.1 mL) were plated onto BHI/LB agar plate for APP and LB agar plate for the other two, incubated for 14 h, and the numbers of bacterial colonies were counted [56].

### ROS detection

The co-cultured cells were washed three times with sterile PBS to remove antibiotics and suspended T cells as mentioned above. The RAW264.7 cells in each group were infected with APP, *K. peumoniae* and *E. coli* (MOI = 5) for 30 min. The uninfected group served as the control, at least three duplicate wells for each group were used. The ROS-specific fluorescent probe 2′,7′-dichlorofluorescein diacetate (DCFH-DA, Beyotime, China) was then used to measure total intracellular ROS levels. DCFH-DA (10 μmol/L) was added after infection, and the cells were incubated for 30 min at 37 °C and 5% CO2. The fluorescence intensity was measured using a fluorescence microplate reader (Tecan, M200 Pro) for APP and flow cytometry (CytoFlex Cytometer, Beckman) for *K. peumoniae* and *E. coli*.

### Detection of dsDNA post APP infection

After co-culture, cells were washed three times with sterile PBS to remove the antibiotics and T cells, and RPMI medium without phenol red was added into each well. After the RAW264.7 cells in each group were infected with APP (MOI = 10) for 30 min, the culture supernatant was collected. Macrophage Extracellular Traps (METs) extraction, purification, and quantitation were performed as previously described [57] using the Quant-iT PicoGreen dsDNA kit (Invitrogen, Carlsbad, CA, USA) for APP and PicoGreen dsDNA Assay Kit (MK bio, China) for *K. peumoniae* and *E. coil*. The fluorescence intensity was measured using a fluorescence microplate reader (Tecan, M200 Pro).

### Statistics

Dunnett test or one-way analysis of variance (ANOVA) or student’s t-test were used to analyze experimental data by GraphPad Prism 9. The data are presented as mean ± standard error of the mean (SEM). Only the statistical difference results were labeled. A *P* value < 0.05 (*) suggests statistical significance; *P* < 0.01 (**) suggests very significant differences; *P* < 0.001 (***) suggests extremely significant differences.

## Supplementary information

Supplementary materials

## Data Availability

The data supporting the findings reported in this study will be available once reasonable request. The mass cytometry data are available via Flow Repository (https://flowrepository.org/id/FR-FCM-Z5CB).
