# Supplementary material for: IL-21-dependent Ly6C+Ly6G+CD4+ T cells found in lung enhance macrophages function against Actinobacillus pleuropneumoniae infection in mice
Source: Cell Death Discov. 2025 Oct 6;11:440. doi: 10.1038/s41420-025-02742-z (PMC12501081; doi:10.1038/s41420-025-02742-z)
Supplement: Supplementary file 1 — Supplementary materials [file 41420_2025_2742_MOESM1_ESM.docx]

**Supplementary Figures**


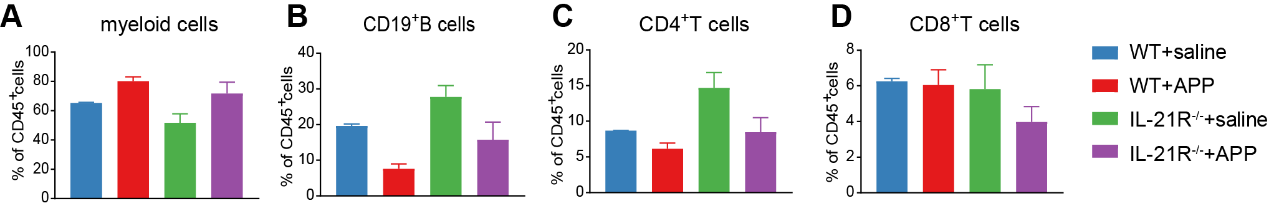


**Fig. S1. Statistical analysis of the proportion of major immune cell lineages in the lung post APP infection at 12 h in each group.**

(A-D) Statistical analysis of the proportion of major immune cell lineages in the lung post APP infection at 12 h in each group. Mann-Whitney U test was used for statistical analysis. Only the statistical difference results were labeled. Error bars show means ± SEM.


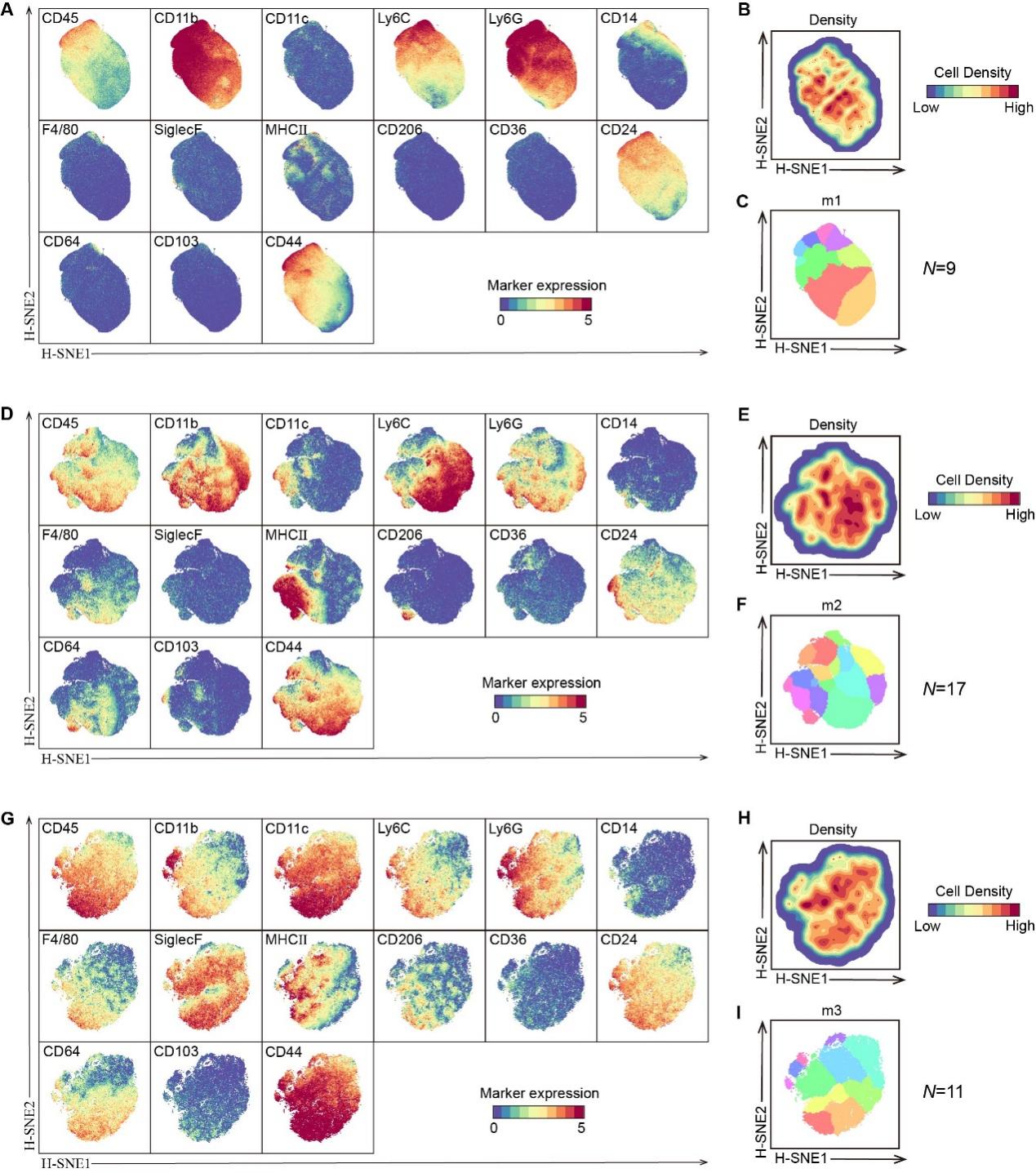


**Fig. S2. t-SNE analysis of myeloid cell metaclusters m1, m2 and m3.**

(A) Expression profiles of the m1 clusters. (B) Density characteristics of cells embedded in m1 group. (C) t-SNE embedding of cells in m1 clusters. Color indicates cluster partitions, m1 was divided into 9 clusters. (D) Expression spectrum of the m1 clusters. (E) Density characteristics of cells embedded in m2 group. (F) t-SNE embedding of cells in m2 clusters. Color indicates cluster partitions, m2 was divided into 17 clusters. (G) Expression spectrum of the m1 clusters. (H) Density characteristics of cells embedded in m3 group. (I) t-SNE embedding of cells in m3 clusters. Color indicates cluster partitions, m3 was divided into 11 clusters.


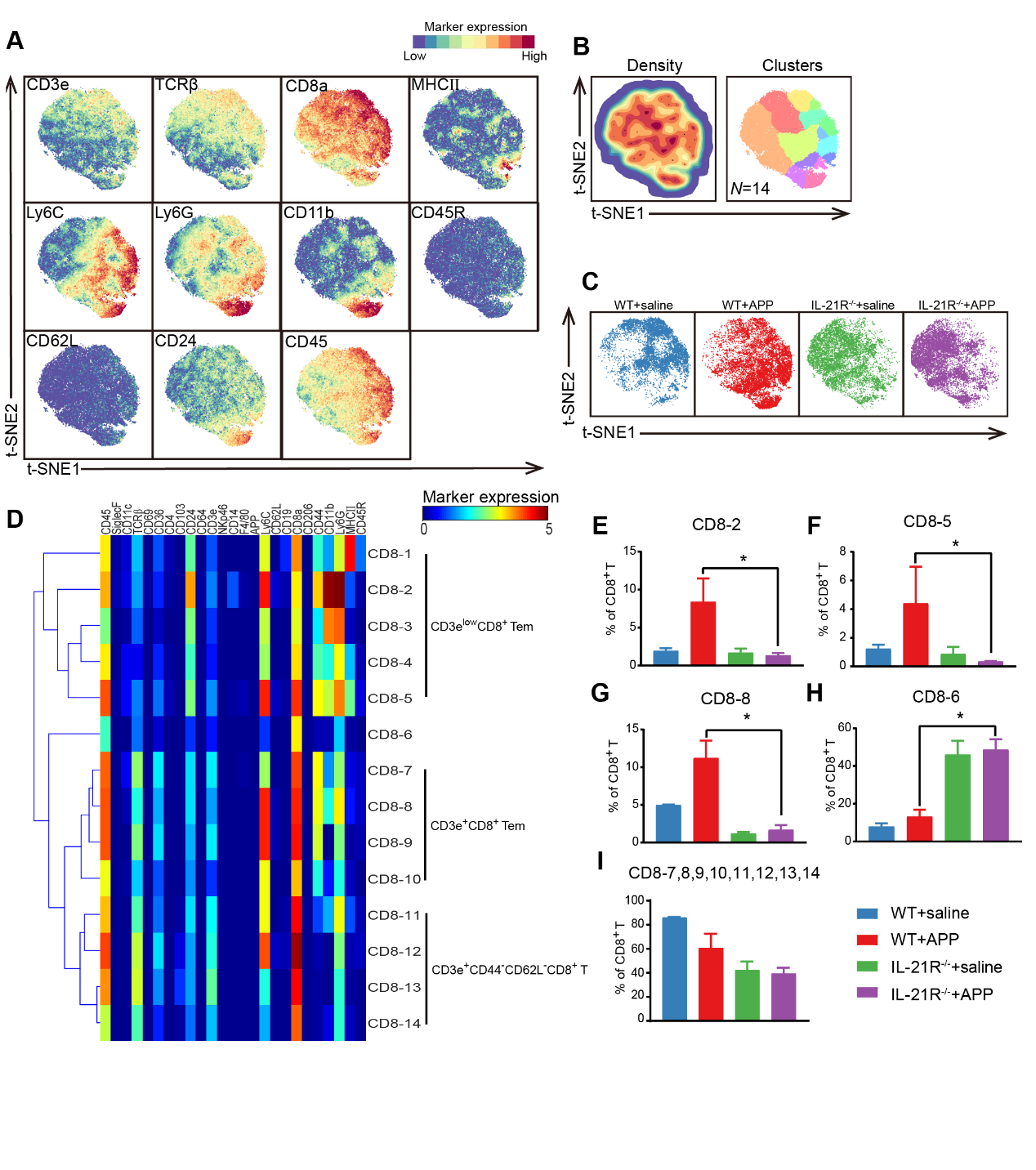


**Fig. S3. Identification of CD8^+^ T lymphocyte clusters in WT and IL-21R^-/-^ mice infected with APP.**

(A) t-SNE embedding of 52,926 CD8^+^ T cells. Colors indicate the expression of the indicated markers. (B) Density map of CD8^+^ T cells. (C) t-SNE plots show the cluster partitions in different colors. (D) Phenotypic heatmap (scale from blue to red) shows the median marker expression values and hierarchical clustering of each cell cluster in CD8^+^ T cells. (E-G) Statistical analysis of the proportion of CD8-2, CD8-5, CD8-8, CD8-6 and CD8-7,8,9,10,11,12,13,14 in CD8^+^ T cells. *p < 0.05, using Mann-Whitney U test. Error bars show means ± SEM.


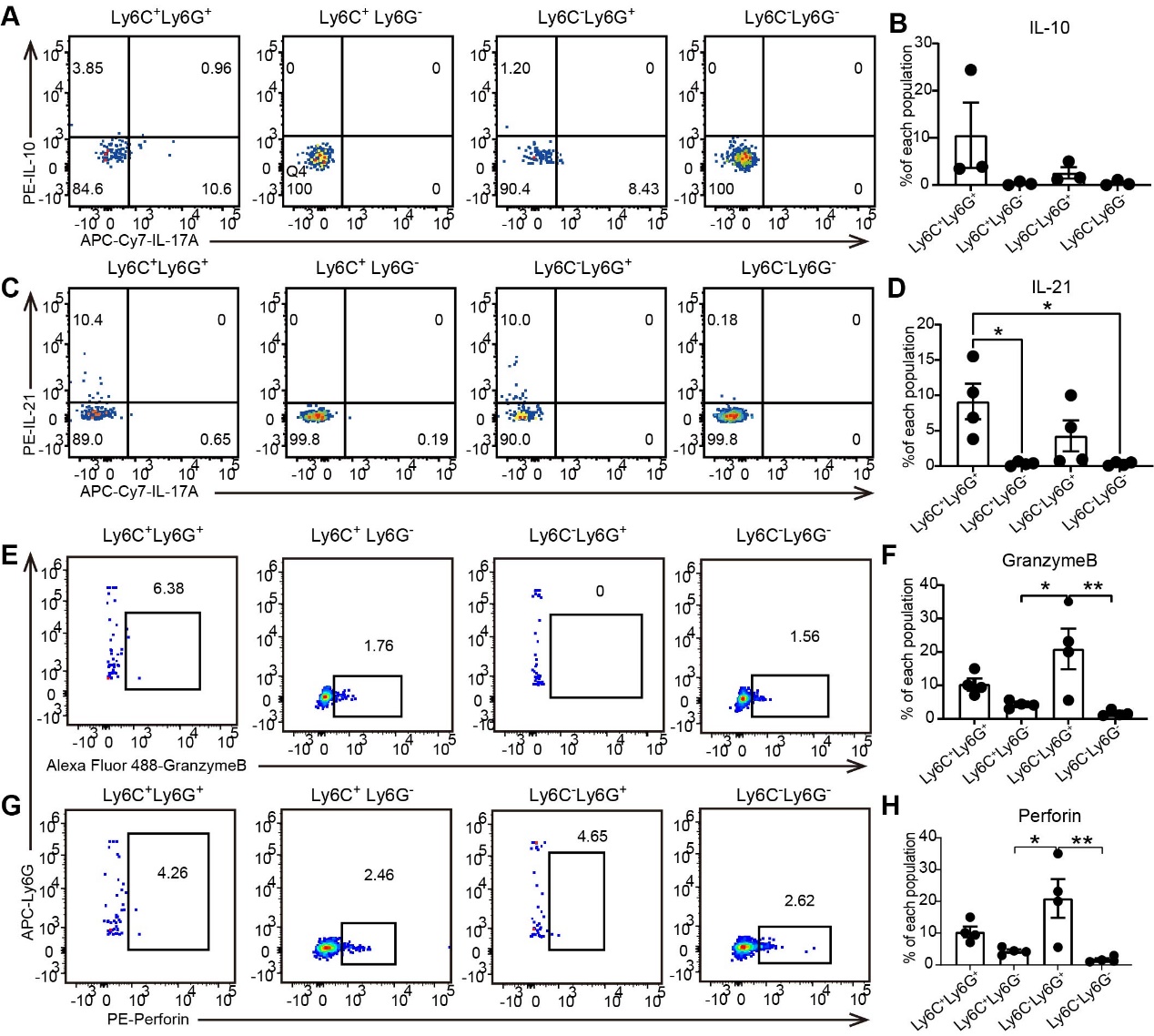


**Fig. S4. Flow cytometric detection of the secretion of IL-10, IL-21, granzyme B and perforin in Ly6C^+^Ly6G^+^CD8^+^ T cells.**

(A-H) Detection and statistics analysis of (A-B) IL-10, (C-D) IL-21, (E-F) granzyme B and (G-H) perforin secretion in Ly6C^+^Ly6G^+^CD8^+^ T cells, Ly6C^+^Ly6G^-^CD8^+^ T cells, Ly6C^-^Ly6G^+^CD8^+^ T cells and Ly6C^-^Ly6G^-^CD8^+^ T cells by flow cytometry. *p < 0.05; **p < 0.01, using Mann-Whitney U test. Error bars show means ± SEM.


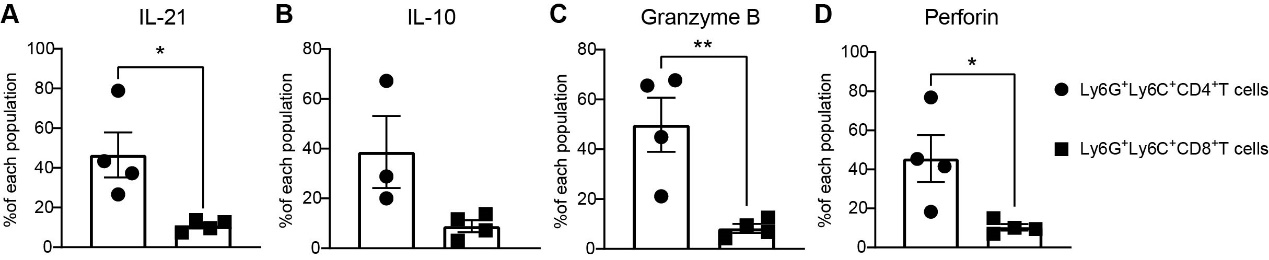


**Fig. S5. Comparison of IL-21, IL-10, granzyme B, and perforin between CD4^+^Ly6C^+^Ly6G^+^T cells and CD8^+^Ly6C^+^Ly6G^+^T cells.**

Statistical analysis of the differences between CD4^+^Ly6C^+^Ly6G^+^T cells and CD8^+^Ly6C^+^Ly6G^+^T cells to secrete (A) IL-21, (B) IL-10, (C) granzyme B, and (D) perforin by flow cytometry. *p < 0.05; **p < 0.01, using unpaired T test. Error bars show means ± SEM.


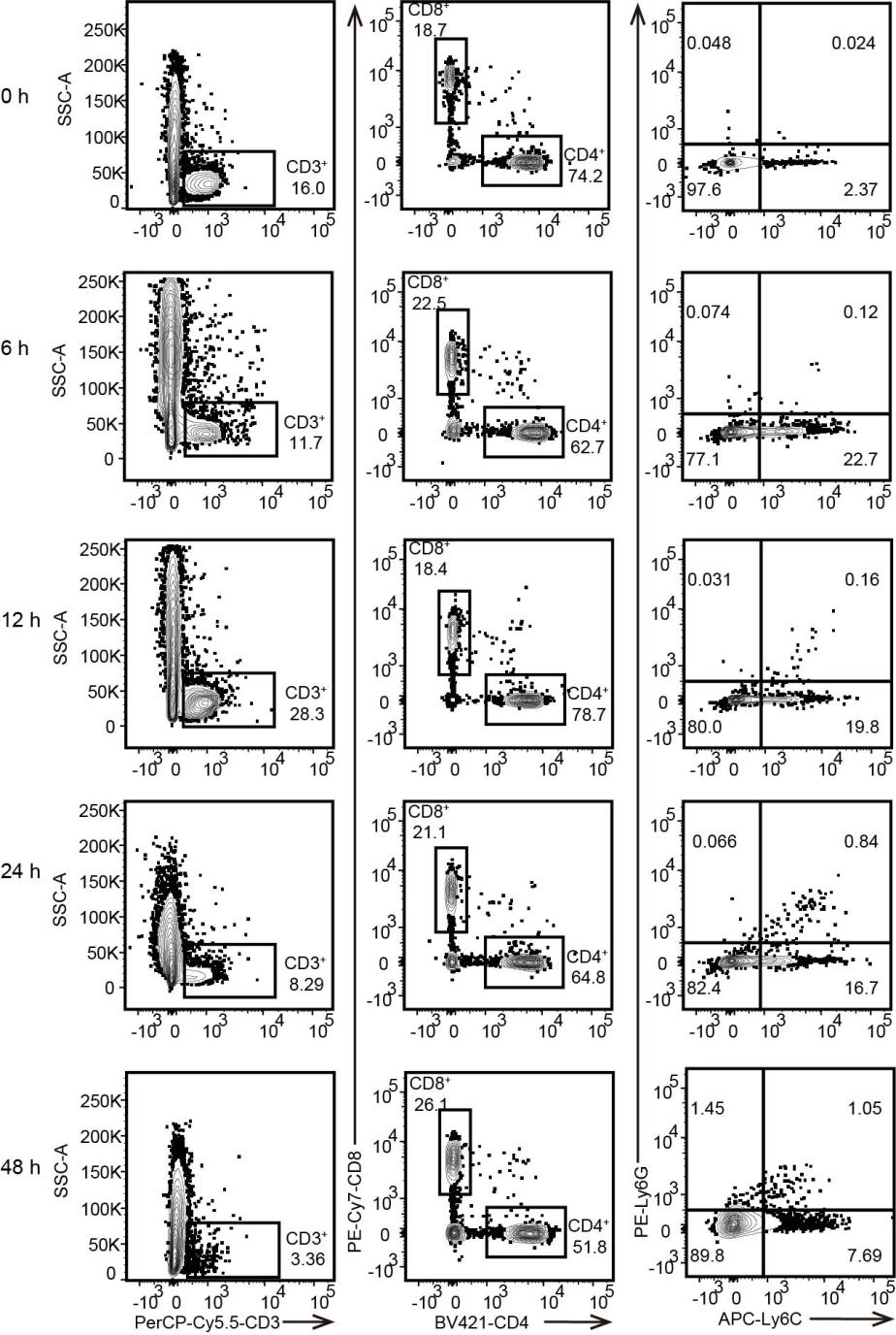


**Fig. S6. The gating strategy for CD4^+^Ly6C^+^Ly6G^+^T cells in the lung of ICR mice infected with *Klebsiella pneumoniae* at 6 h, 12 h, 24 h, 48 h and without infection (0 h), as an example.**


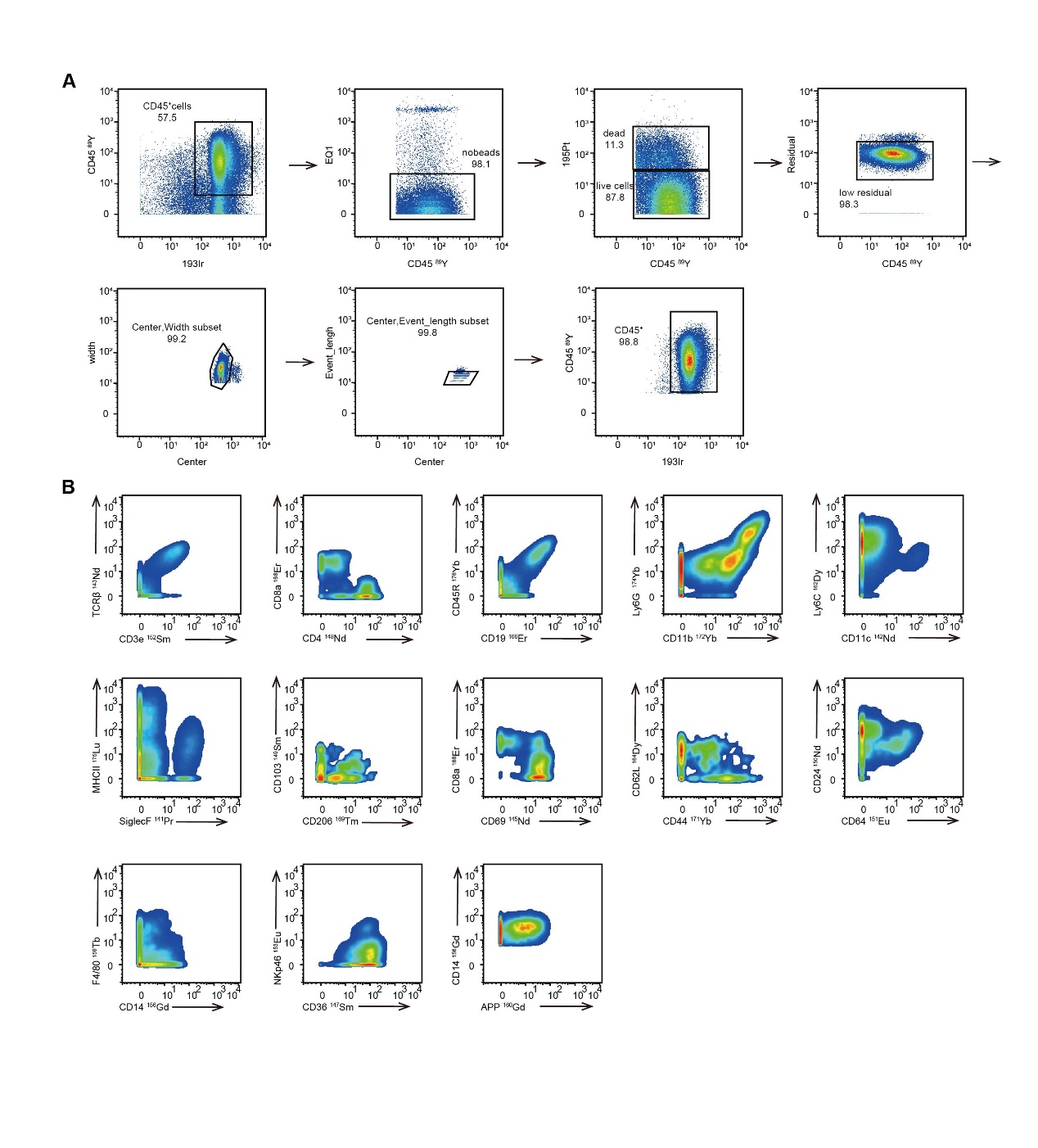


**Fig. S7. Gating strategy and validation of antibody used in mass cytometry on CD45^+^ single, live cells in mouse lung.**

(A) Gating strategy of single CD45^+^ live cells in mouse lung. (B) Biaxial plots show the indicated marker expression on lung CD45^+^ immune cells.

**Supplementary Table 1. Primers used for RT-qPCR**

| Gene name | Forward primer (5’ – 3’) | Reverse primer (5’ – 3’) |
| --- | --- | --- |
| *IL-21* | AAGATTCCTGAGGATCCGAGAAG | GCATTCGTGAGCGTCTATAGTGTC |
| *IL-10* | CTTACTGACTGGCATGAGGATCA | GCAGCTCTAGGAGCATGTGG |
| *TGF-β* | CGAAGCGGACTACTATGCTAAAGAG | TGGTTTTCTCATAGATGGCGTTG |
| *IFN-γ* | CTCTTCCTCATGGCTGTTTCT | TTCTTCCACATCTATGCCACTT |
| *Actin* | AGCCTTCCTTCTTGGGTATG | GTGTTGGCATAGAGGTCTTTAC |

**Supplementary Table 2. Antibodies information used for mass cytometry**

| **Marker** | **Catalog No.** | **Source** | **Clone number** | **Metal isotope** |
| --- | --- | --- | --- | --- |
| CD45 | 3089005B | Fluidigm | 30-F11 | 89Y |
| SiglecF | 155502 | Biolegend | S17007L | 141Pr |
| CD11c | 3142003B | Fluidigm | N418 | 142Nd |
| TCRβ | 3143010B | Fluidigm | H57-597 | 143Nd |
| CD69 | 3145005B | Fluidigm | H1.2F3 | 145Nd |
| CD36 | 3147013B | Fluidigm | No.72-1 | 147Sm |
| CD4 | 100561 | Biolegend | RM4-5 | 148Nd |
| CD103 | 121401 | biolegend | 2E7 | 149Sm |
| CD24 | 3150009B | Fluidigm | M1/69 | 150Nd |
| CD64 | 3151012B | Fluidigm | X54-5/7.1 | 151Eu |
| CD3e | 3152004B | Fluidigm | 145-2C11 | 152Sm |
| NKp46 | 3153006B | Fluidigm | 29A1.4 | 153Sm |
| CD14 | 3156009B | Fluidigm | Sa14-2 | 156Gd |
| F4/80 | 3159009B | Fluidigm | BM8 | 159Tb |
| Ly6C | 3162014B | Fluidigm | HK1.4 | 162Dy |
| CD62L (L-selectin) | 3164003B | Fluidigm | MEL-14 | 164Dy |
| CD19 | 3166015B | Fluidigm | 6D5 | 166Er |
| CD8a | 3168003B | Fluidigm | 53-6.7 | 168Er |
| CD206 (MMR) | 3169021B | Fluidigm | C068C2 | 169Tm |
| CD44 | 3171003B | Fluidigm | IM7 | 171Yb |
| CD11b (Mac-1) | 3172012B | Fluidigm | M1/70 | 172Yb |
| Ly6G | 3141008B | Fluidigm | 1A8 | 174Yb |
| MHCII | ab55152 | Abcam | 6C6 | 175Yb |
| CD45R (B220) | 3176002B | Fluidigm | RA3-6B2 | 176Yb |
| APP | Made in house Polyclone | | | 160Gd |

**Supplementary Table 3. Antibodies information used for flow cytometry**

| **Marker** | **Catalog No.** | **Source** | **Clone number** | **Fluorescein** |
| --- | --- | --- | --- | --- |
| CD3 | 100203 | Biolegend | 17A2 | FITC |
| CD4 | 100437 | Biolegend | GK1.5 | BV421 |
| CD8a | 100722 | Biolegend | 53-6.7 | PE/Cy7 |
| Ly6C | 128033 | Biolegend | HK1.4 | BV510 |
| Ly6G | 46-9668-88 | ebioscience | 1A8 | PerCP-eFlour710 |
| Ly6G | 127614 | Biolegend | 1A8 | APC |
| CD3 | 100217 | Biolegend | 17A2 | PerCP-Cy5.5 |
| IL-21 | 12-7211-82 | ebioscience | FFA21 | PE |
| IFN-γ | 505810 | Biolegend | XMG1.2 | APC |
| TNF-α | 506307 | Biolegend | MP6-XT22 | APC |
| IL-6 | 504507 | Biolegend | MP5-20F3 | APC |
| IL-17A | 506940 | Biolegend | TC11-18H10.1 | APC/Cy7 |
| Perforin | 154306 | Biolegend | S16009A | PE |
| Granzyme B | 372206 | Biolegend | QA16A02 | FITC |
| CD86 | 105014 | Biolegend | GL-1 | PE/CY7 |
| CD206 | 141708 | Biolegend | C068C2 | APC |
| CXCR5 | 145503 | Biolegend | L138D7 | PE |
| CD3 | 953316 | Southernbiotech | PPT3 | FITC |
| CD4 | 561474 | BD | 27367 | PerCP-Cy5.5 |
| CD8 | 957242 | Southernbiotech | 27801 | APC |
| CD14 | FAB4597N | RD | 433423 | APC/A700 |
